# Development of a Sensory Neuron-Integrated Skin Spheroid Model for the Evaluation of Neuropeptide-Based Topical Delivery Systems

**DOI:** 10.1021/acsbiomaterials.5c00141

**Published:** 2025-05-23

**Authors:** Bianca Aparecida Martin, Juliana Viegas, Luciana Facco Dalmolin, Emerson de Souza Santos, Izabela Pereira Vatanabe, Sabrina Francesca Lisboa, Renata Fonseca Vianna Lopez, Bruno Sarmento

**Affiliations:** † School of Pharmaceutical Sciences of Ribeirão Preto, 67782 University of São Paulo, Avenida do Café, s/n°, 14040-903 Ribeirão Preto, São Paulo, Brazil; ‡ i3S - Instituto de Investigação e Inovação em Saúde, Universidade do Porto, Rua Alfredo Allen 208, 4200-135 Porto, Portugal; § 92905 INEB - Instituto de Engenharia Biomédica, Universidade do Porto, Rua Alfredo Allen 208, 4200-135 Porto, Portugal; ∥ IUCS-CESPU - Instituto Universitário de Ciências da Saúde, Rua Central de Gandra 1317, 4585-116 Gandra, Portugal

**Keywords:** aged skin, acetyl hexapeptide-3, skin spheroids, neuronal model, aging products

## Abstract

The skin is a complex organ composed of multiple layers and diverse cell types, including keratinocytes, fibroblasts, adipocytes, and sensory neurons, which maintain its structural and functional integrity together. Conventional in vitro and ex vivo models help investigate drug permeation and selected biological effects. However, they are limited in replicating neural interactions critical for assessing the efficacy of neuropeptide-based therapies. To address this limitation, a sensory neuron-integrated skin spheroid (SS) model was established, incorporating key skin cell types and providing a rapid, adaptable, and physiologically relevant platform for screening the biological activity of topical delivery systems targeting neuronal pathways. The model’s responsiveness was demonstrated using acetyl hexapeptide-3 (HEX-3), a neuropeptide that inhibits acetylcholine release. HEX-3 was internalized by spheroid cells, with preferential accumulation around sensory neurons, confirming targeted cellular uptake. In parallel, ex vivo human skin studies confirmed that HEX-3 can traverse the stratum corneum and accumulate in deeper layers. Treatment with this film enhanced skin hydration, reduced scaling, and improved the structural organization of the stratum corneum after 48 h. Functional assays using the SS model showed that HEX-3 treatment suppressed acetylcholine release, upregulated the antioxidant enzyme SOD2, and stimulated type I collagen synthesis. In aged skin samples, the application of HEX-3 significantly increased collagen levels. This effect was mirrored in the spheroid model, which reached collagen levels comparable to those of aged human skin upon treatment. These findings establish the SS model as a robust platform for evaluating the biological activity of neuropeptide-based topical therapies, offering valuable insights for developing advanced strategies for skin rejuvenation and repair.

## Graphical abstract

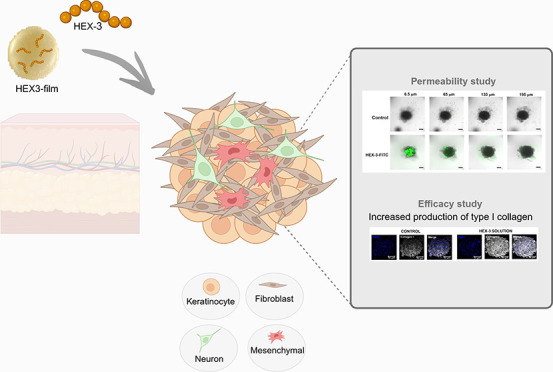

## 1 Introduction

The skin is one of the most complex organs in the human body, consisting of three distinct layers: the epidermis, dermis, and hypodermis. The outermost epidermis is composed primarily of keratinocytes at various stages of differentiation, organized into four sublayers: the basal, spinous, granular layers, and the stratum corneum (SC). The SC, formed by dead, keratinized cells, is the principal barrier, shielding the body from environmental aggressors. Beneath the epidermis lies the dermis, a connective tissue-rich layer populated predominantly by fibroblasts. Separated from the epidermis by the basement membrane, the dermis houses sebaceous and sweat glands, blood vessels, cutaneous nerve fibers, and sensory receptors. This layer is embedded in an extracellular matrix (ECM) composed mainly of type I and III collagen, secreted by fibroblasts, critical for maintaining skin firmness and elasticity. The innermost hypodermis comprises epithelial and adipose cells, providing mechanical protection and contributing to thermoregulation.^1^

Despite this structural complexity, the skin remains a widely exploited route for noninvasive drug delivery, owing to its large surface area and accessibility. Drugs can be applied topically to achieve local effects or transdermally for systemic absorption. Various in vitro and ex vivo models have been established to study drug penetration and biological effects to support the development of such strategies. The most commonly used models for evaluating drug penetration involve excised human skin, typically obtained from surgical procedures, or porcine skin.^2,3^ As the SC constitutes the main barrier and is composed of nonviable cells, maintaining viable deeper layers is often unnecessary in these models, allowing the use of frozen tissues. These models are therefore particularly valuable for assessing how formulations modulate skin permeability.

However, when the aim is to evaluate drug toxicity or prodrug metabolism, which require viable cells, more sophisticated models are needed. Reconstructed human skin models offer an advanced alternative, recapitulating native skin architecture with organized layers and, in some cases, incorporating additional elements such as melanocytes, immune cells, or neurons.^4,5^ While biologically more relevant than frozen skin, their SC barrier function does not fully replicate that of surgically excised human skin, limiting their suitability for penetration studies. Additionally, these models are expensive, require several weeks for construction and differentiation, and often fail to reproduce complex inflammatory or neural responses.

As a faster and more adaptable alternative, skin spheroid models have been proposed for formulation screening and the investigation of specific biological activities.^6^ Although these models do not replicate the layered architecture of native skin and are limited in evaluating barrier properties such as vertical diffusion, they are inexpensive, easy to assemble, and enable the rapid inclusion of diverse cell types, such as keratinocytes, fibroblasts, and adipocytes, to create tailored microenvironments. Nevertheless, no spheroid models incorporating sensory neurons have been reported to evaluate the activity of topically applied neuromodulators.

Several bioactive compounds targeting sensory neurons are currently employed to improve the appearance of aged skin, which undergoes cellular and ECM alterations that compromise its structural and functional integrity.^7^ Among these, neuropeptide-based therapies such as botulinum toxin and acetyl hexapeptide-3 (HEX-3) are widely used. For effective action, these agents must first traverse the SC, a property assessable using standard surgical skin models, and subsequently reach deeper layers to interact with neuronal targets. However, more appropriate skin models are required to determine whether such interactions result in the desired biological effects.

In this study, we present the development of an advanced sensory skin spheroid model comprising keratinocytes, fibroblasts, adipocytes, and sensory neurons for the first time. To validate the model’s responsiveness to neuropeptide-mediated activity, spheroids were treated with HEX-3, a neuropeptide known to inhibit acetylcholine release.^8^ Furthermore, to assess the robustness of the spheroids under exposure to topical delivery systems, we engineered a polymeric film for HEX-3 delivery. This film, composed of silk fibroin and hyaluronic acid (HA), was previously demonstrated by our group to be biocompatible in its unloaded form.^9^ In the present study, the HEX-3-loaded film was comprehensively characterized in terms of its physicochemical properties, peptide release kinetics, biocompatibility, and ex vivo skin response. To further substantiate the utility of our spheroid model for assessing biological responses elicited by topical systems, treated spheroids were evaluated for their capacity to suppress acetylcholine release, enhance antioxidant defenses through SOD2 gene upregulation, and stimulate collagen type I synthesis.

## 2 Experimental Section

### 2.1 Materials

Trypsin-EDTA 0.25% (Gibco); Dulbecco’s Modified Eagle Medium – DMEM (Biowest); penicillin-streptomycin (Gibco); fibroblasts medium (Innoprot); heat inactivated fetal bovine serum (LabClinics S181BH-500); Alamar-Blue (Resazurin assay – Thermo Fisher Scientific); Non-Essential Amino Acids 100 (Gibco, 11140); microtissue molds 3D Petri Dish (Merck); formaldehyde 20% solution (Electron Microscopy Sciences (EMS)); Trypan Blue 0.4% solution (Gibco); OCT embedding matrix (VWR); CellTiter-Glo (Promega); Gill’s hematoxylin solution (Lusoplex); alcoholic eosin (Lusoplex); Entellan mounting medium and dimethyl sulfoxide (DMSO) (Merck); Masson Trichrome (Epredia, Thermo Fisher Scientific); Triton X-100 (Thermo Fisher Scientific A16046-AP); Fluoromount (Merck); sodium chloride anhydrous; Neural Factor Growth (NGF-β) from rat (Sigma-Aldrich); Choline/Acethylcholine Quantification Kit (Sigma-Aldrich); DAPI (4′,6′-diamino-2-Fenil-indol) (Merck D9542); fluorescein isothiocyanate (Fluka, 46950); chitosan oligosaccharide lactate (average Mn 5000 g/mol, >90% deacetylated) (chitosan) (Sigma-Aldrich); hyaluronic acid sodium salt (HA) from *Streptococcus equi* (MW ∼ 1.5–1.8 × 10^6^ Da) (Sigma-Aldrich); poly­(vinyl alcohol) (PVA) (87–90% hydrolyzed, MW 30–70 kDa) (Sigma-Aldrich); AllPrep DNA/RNA/Protein Mini Kit (Qiagen); mesenchymal stem cells medium (Innoprot); DMEM/F12, F12K and Horse serum (Gibco); SeaKem LE Agarose (Lonza); antibody primary β-tubulin III (Promega Corporation); NeuN (Abcam); chloromethyl-2′,7′-dichlorodihydrofluorescein diacetate (CM-H_2_DCFDA [850013-49-9]) (Quimigen); Corneofix strips (Fisher Chemical); and deionized water (18.2 MΩ^–1^ cm^–1^ at 25 °C) (Milli-Q, Direct-Q 3 UV, Millipore). HEX-3 was purchased from MedChemExpress (USA), and silk fibroin cocoons produced by the silkworm (*Bombyx mori*) were obtained from “O Casulo Feliz” (Maringá, PR, Brazil).

### 2.2 Methods

#### 2.2.1 Cellular Culturing Conditions

HaCaT (Human keratinocytes) and NSC-34 (motor neurons) were cultured and expanded in DMEM High Glucose (4.5 g/L) medium, supplemented with 1% (v/v) penicillin/streptomycin solution (100 U/mL penicillin and 100 μg/mL streptomycin), 10% (v/v) fetal bovine serum (FBS), and 1% (v/v) nonessential amino acids (MEM-NEAA). Human dermal fibroblasts (HDF) were cultured and expanded in fibroblast medium supplemented with 1% penicillin/streptomycin solution, fibroblast growth factor, and 1% (v/v) FBS. Human mesenchymal stem cells (hMSCs) were cultured and expanded in hMSC medium, supplemented with 1% (v/v) penicillin/streptomycin solution, hMSC growth supplements, and 5% (v/v) FBS. PC12 Adh (pheochromocytoma of the adrenal medulla of rat) were cultured and expanded in F12K medium, supplemented with 15% (v/v) horse serum (HS), 2.5% (v/v) FBS and 1% (v/v) penicillin/streptomycin. All cells were incubated at 37 °C in a atmosphere with 5% CO_2_ and 95% relative humidity.

#### 2.2.2 Neuronal Differentiation Procedure

NSC-34 cells were seeded in 6-well plates at a density of 2 × 10^5^ cells per well, and after 24 h, 2 mL of differentiation medium (DMEM/F12 supplemented with 1% FBS, 1% MEM-NEAA, 1% penicillin/streptomycin solution, and 1 μM retinoic acid) was added to each well and incubated for 4 days. The retinoic acid (RA) concentration was optimized in prior experiments.

Subsequently, to confirm the differentiation, besides morphological imaging (Zoe microscope), the β-tubulin III marker of neurons-like cells were used. The differentiated cells were seeded onto coverslips which were pretreated with 100 μL of poly-l-lysine (100 μg/mL) for 30 min. The same protocol for NSC-34 differentiation was applied. Then, the cells were fixed with 4% (v/v) paraformaldehyde (PFA) for 20 min at room temperature (RT), permeabilized with 5% FBS + 0.25% Triton X-100 in phosphate-buffered saline (PBS, 150 mM, pH 7.4), and blocked with 500 μL of 5% (v/v) FBS. The cells were incubated overnight at 4 °C with the primary antibody, antimouse β-tubulin III (1:2000). Following this, the cells were incubated for 1 h with the secondary antibody, Alexa Fluor 647 (antimouse). After incubation with secondary antibody, the coverslips were washed twice with PBS and incubated with DAPI (4′,6-diamidino-2-phenylindole) (500 ng/mL) for nuclei staining and Alexa Fluor 488 Phalloidin (1:2000) for labeling cytoskeleton, for 20 min. They were washed again twice with PBS and mounted on slides using Fluoromount aqueous mounting medium. Images were captured in a Confocal microscope, using a 40× oil immersion objective lens.

PC12 Adh cells at the density 5 × 10^5^ cell/well were seeded in 6 well plate, and treated with differentiated culture medium, F12K supplemented with 1% (v/v) HS, 1% (v/v) penicillin/streptomycin solution, and NGF-β at 100 ng/mL for 8 days to obtain differentiated neurons. The medium was changed every 2 days. Subsequently, besides morphological imaging, the NeuN marker of neuron-like cells was used to confirm the differentiation. The differentiated cells (3 × 10^4^ cells/well) were seeded onto coverslips, which were pretreated with 100 μL of poly-l-lysine (100 μg/mL) in the 24-well plate for 30 min. The same protocol for PC12 differentiation was applied. Then, the cells were fixed with 4% (v/v) PFA for 10 min at room temperature, permeabilized with 0.1% Triton X-100 in PBS, and blocked with 500 μL of 1% of bovine serum albumin (p/v) with 0.1% of Tween 20 (20% in PBS). The cells were incubated overnight at 4 °C with the primary antibody, antirabbit NeuN (1:500). Following this, the cells were incubated for 1 h with the secondary antibody, Alexa Fluor 594 (antirabbit). After incubation, they were washed twice with PBS and incubated with DAPI (4′,6-diamidino-2-phenylindole) (1:1000) for nuclei staining and Alexa Fluor 488 Phalloidin (1:225) for labeling the cytoskeleton, for 20 min. They were washed again twice with PBS and mounted on slides using Fluoromount aqueous mounting medium. Images were captured in a Confocal microscope, using a 63× oil immersion objective lens.

#### 2.2.3 Ultrastructure Characterization of Differentiated Neuronal Cells

The pellet of differentiated NSC-34 was stained overnight at 4 °C, in a 2% (v/v) osmium tetroxide solution in 0.1 M sodium cacodylate. After staining, the cells were washed thrice for 10 min with 0.1 M cacodylate. To improve the microscopic visualization of the cells, they were transferred to 1% (v/v) uranyl acetate and incubated for 1 h at 4 °C in the dark. Then, they were immobilized in Histogel and dehydrated in a gradient series of ethanol and propylene oxide solutions for 10 min each. Thus, the cells were included in EPON resin by gradual immersion of an increasing series of propylene oxide to EPON (3:1, 1:1, 1:3, and 0:1) for 1 h each. After that, EPON resin was included in a silicon mold. EPON polymerization was incubated at 60 °C for 48 h. Sections with 60 nm thickness were prepared using a diamond knife (Diatome, Hatfield, PA, USA) and were recovered to 200 mesh Cu-grids. Staining of sections using 2% uranyl acetate (w/v) was performed before observation. The images were acquired at 80 kV in a Jeol JEM-1400 transmission electron microscope (Japan) with a CCD digital camera Orious 1100 W (Tokyo, Japan).

#### 2.2.4 Sensory Spheroids Assembly and Characterization

##### 2.2.4.1 Microtissue Molds Assembly Technique

Sensory spheroids (SS) were produced using micromolds (3D Petri Dish, MicroTissues Inc.). Briefly, the molds were made using agarose (2%, w/v) dissolved in sodium chloride (0.9%, w/v). They were placed in 24-well plates 2 h before SS seeding. First, different cell densities were used to standardize the best conditions for producing SS. A cell suspension (65 μL), containing 4000, 6000, 8000, or 9000 cells per spheroid, was added into the molds and left undisturbed for 30 min to allow the cells to deposit into the molds’ wells. Afterward, the wells were filled with 1 mL of DMEM. The spheroids were cultured for up to 10 days, with medium changes every 2 days.

Controls of spheroids were produced in monoculture (HaCaT and HDF separately) with four different cell densities (2000; 3000; 4000; and 6000 cell per spheroid) and in coculture (HaCaT and HDF) with nine different cell ratios: 1:1 (4000; 6000; and 8000 cells per spheroid), 1:2, and 2:1 (6000 and 9000 cells per spheroid), and 1:3 and 3:1 (8000 cells per spheroid). Subsequently, three selected conditions underwent histological analysis based on metabolic activity, spherical shape, and cell compaction.

Finally, the selected coculture condition, called epidermal–dermal spheroid (EDS), at a ratio of 1:2 (9000 total cells per spheroid), received 5% of the coculture population of differentiated neuronal cells and human mesenchymal stem cells (hMSCs) (450 cells of each). These cells were added to simulate nerve connection-skin, and the subcutaneous layer of the skin, respectively. This resulted in sensory-enhanced skin spheroids, a quadruple spheroid model called SS. Thus, the SS was determined for further experiments as a 1:2:0.15:0.15 ratio (9900 total cells per spheroid) of fibroblasts, keratinocytes, differentiated neuronal cells, and mesenchymal stem cells (adipocyte progenitors), respectively.

##### 2.2.4.2 Diameter and Metabolic Activity

The EDS and SS were followed over time using a ZOE Fluorescent Cell Imager (Bio-Rad Laboratories, California, USA), and images were captured to analyze the relative diameter. The average diameter of each spheroid was determined by averaging two axes (horizontal and vertical) using ImageJ NIH software. Ten spheroids from each condition were evaluated. At the same time, the metabolic activity was assessed by CellTiter-Glo reagent, following the manufacturer’s protocol. Briefly, three spheroids from each condition were collected from the molds and individually transferred to a 96-well plate. 100 μL of CellTiter-Glo was added to each spheroid in 100 μL DMEM, followed by an incubation for 30 min at room temperature. Luminescence was measured using a Synergy 2 microplate reader (BioTek, USA).

##### 2.2.4.3 Histological Analysis

For histological analysis, it was selected, based on metabolic activity and roundness, the monoculture spheroids HDF of 5000 cells and HaCaT of 5000 cells, also the EDS 1:1 (8000 cells per spheroid), 1:2 (9000 cells per spheroid), and 1:3 (8000 cells per spheroid). In addition, histology was also performed for the SS on days 4 and 7 in the culture. Briefly, the DMEM was removed from the molds; the spheroids were fixed with PFA 4% (v/v) for 20 min. The samples were washed once with PBS 1×, and then agarose (1%, w/v) was added to close the molds. The molds were transferred to histological cassettes for histological procedures. The processing was made by dehydrating the samples in a graded alcohol series starting from 50% to absolute, following xylene and then paraffin. After embedding, the samples were sectioned into 4 μm slices using a Leica RM2255 microtome (Wetzlar, Germany) and stained with hematoxylin and eosin (H&E). Images captured using a Brightfield microscope (Leica DM2000 LED, Wetzlar, Germany).

##### 2.2.4.4 Immunofluorescence

Paraffin sections were dewaxed in xylene and rehydrated in a graded alcohol series of decreasing concentrations, and then they were incubated in sodium citrate buffer (0.01 M, pH 6) for antigen retrieval for 30 min, at 96 °C. Samples were permeabilized with 0.25% Triton X-100 and blocked with 10% FBS (v/v) in PBS 1× for 1 h at RT. After permeabilization and blocking, the slices were incubated with primary antibodies for type I collagen, vimentin, fibronectin, β-tubulin III, and choline acetyltransferase (ChAT) in a wet chamber at 4 °C overnight. After that, the slices received the secondary antibody, incubating for 1 h at room temperature. Nuclei were stained with 4′,6-diamidino-2-phenylindole (DAPI) (0.5 μg/mL), and the cytoskeleton was stained with Alexa Fluor 546 Phalloidin diluted in 5% (v/v) FBS. The specifications of primary and secondary antibodies are listed in the Supporting Information, Table S1.

#### 2.2.5 Antiaging Polymeric Films Preparation and Characterization

##### 2.2.5.1 Polymers Preparation

Silkworm cocoons (*Bombyx mori*) were cleaned, cut into small pieces, and added to aqueous 0.5% sodium carbonate solution at 70 °C (1:200 silk fibroin/solvent, w/v). This mixture was stirred for 60 min with solvent changes every 20 min to remove the sericin. The obtained silk fibroin fibers were washed twice with purified water and dried at room temperature for 2 days. The dried silk fibroin fibers were then dispersed in a ternary calcium chloride solution:ethanol (1:2:8 mol) (1:10 w/v fibroin/solvent) and incubated in a water bath at 60 °C overnight. The dispersion was filtered through gauze and dialyzed using a cellulose acetate membrane (3500 Da) in ultrapure water for 72 h. The dialysis water was changed every 24 h until the conductivity of the dialysis solution matched that of ultrapure water. The concentration of solubilized silk fibroin in the dialyzed dispersion was determined based on the measurement of solid residue percentage using a moisture analyzer (Ohaus, Barueri, SP, Brazil) and ranged from 3.5 to 4%.^10^ Hyaluronic acid (HA) (5 mg/mL) was dispersed in ultrapure water and stirred overnight at room temperature. The dispersion was then subjected to ultrasound at 20 kHz for 1 min with an amplitude of 40%. Chitosan, PVA, NaCl, and HEX-3 were separately prepared in ultrapure water at 37.5, 50, 5, and 1 mg/mL, respectively.

##### 2.2.5.2 Film Casting

The polymeric films containing HEX-3 were prepared using the film casting (solvent evaporation) technique.^9,11^ A dry film surface of 25 mg/cm^2^ was prepared according our previous paper^9^ to contain 70% of a silk fibroin and chitosan mixture (1:1), 3.5% HA, 3.5% PVA, 16% glycerol, and 750 ng/cm^2^ of the HEX-3 peptide. For film preparation, the silk fibroin dispersion and an aqueous chitosan solution were prepared separately, and then equal parts of the aqueous PVA solution were added. The HA dispersion (Section 2.2.5.1) was added to the silk fibroin/PVA dispersion and mixed with the chitosan/PVA. Finally, glycerol, and HEX-3 solubilized in water (750 ng/cm^2^) were added, and the final mixture, approximately 1.7 mL, was poured onto a Teflon plate (1.77 cm^2^) and dried in an incubator (Eletrolab, EL101/4, São Paulo, Brazil) at 35 °C and 40% relative humidity for approximately 24 h. Films without HEX-3 (Blank films) were obtained as controls.

##### 2.2.5.3 Films Thermal and Chemical Analysis

A differential scanning calorimeter (PerkinElmer, Jade DSC, Massachusetts, USA) was used for differential scanning calorimetry (DSC) analysis under a nitrogen flow of 3 kgf/cm^2^. The system was equilibrated at 20 °C for 1 min and then heated linearly to 250 °C at a heating rate of 5 °C/min. The mass of the samples analyzed was 2 mg. Fourier Transform Infrared Spectroscopy (FTIR) analyses of the films were performed in attenuated total reflectance (ATR) mode in the range of 4000–500 cm^–1^ using an FTIR spectrometer (Bruker, Tensor 27, USA).

##### 2.2.5.4 Swelling and Mechanical Analysis

The films’ swelling behavior and mechanical properties were assessed to evaluate their structural integrity and potential application as bioadhesive materials. The gravimetric method determined the degree of swelling (%), representing the percentage of liquid absorbed by the films. Films with or without HEX-3 (0.5 × 1.0 cm) were placed in a cylindrical device with a nylon mesh (138 × 75 μm opening) and submerged in 10 mL of PBS within a polypropylene plate. The initial mass of the films was recorded before immersion in PBS. The films were weighed at intervals over 72 h, with excess buffer solution removed before each measurement. The swelling percentage was calculated as the difference between the initial and final mass, relative to the initial film weight.

The mechanical properties were evaluated using a texture analyzer (TA.XT.PLUS, Stable Micro System Co. Ltd., England). Films (5 × 11 mm) were vertically oriented at 25 °C, with their ends coated in adhesive tape to prevent separation and precisely fixed in the center of the grips. Tensile strength (MPa) was determined by dividing the breaking force (N) by the cross-sectional area of the film, while elongation at break (%) was calculated based on the differential rupture extension relative to the initial grip separation. Young’s modulus (MPa) was derived by dividing the tensile force by the elongation at break. Moreover, the bioadhesive property was evaluated using the tension mode. Human skin samples were prehydrated in PBS for approximately 10 min and then, they were placed on the lower part of the equipment, with a drop of cyanoacrylate to ensure fixation. Prehydrated films (1.13 cm^2^), containing or not HEX-3, were attached to the upper probe with double-sided tape. The probe was lowered until it touched the skin surface, maintaining contact with a force of 0.5 N for 3 min. After this period, the probe was raised at a constant speed of 1 mm/s until the sample separated from the skin. The force required to detach the film from the skin (detachment force) and the work of bioadhesion (area under the force vs distance curve) were determined using Exponent software (Stable Micro Systems, UK).

##### 2.2.5.5 Microscopical Morphology

The films were analyzed by scanning electron microscopy (SEM) using a Shimadzu SS-550 microscope (Shimadzu, Kyoto, Japan) with an electron beam acceleration voltage of 20 kV for cross-sectional analyses and 5 kV for surface analyses. An Everhart-Thornley detector was used to collect secondary electrons emitted from the sample. The samples were mounted horizontally and vertically on aluminum sample holders using carbon-based double-sided conductive tape (Ted Pella Inc., California, USA). Subsequently, the samples were coated with gold (Bal Tec, SCD 050 Sputter Coater, Fürstentum, LI) for 120 s to ensure electrical conductivity of their surfaces for morphological analysis.

Atomic force microscopy (AFM) (Shimadzu, SPM-9600, Kyoto, Japan) with silicon tips (Nanosensors, PPPNCHR, Neuchâtel, Switzerland) and the following cantilever specifications: thickness: 4.0 ± 1 μm, length: 125 ± 10 μm, width: 30 ± 7.5 μm, resonance frequency: 204–497 kHz, force constant: 10–130 N/m, and tip height: 10–15 μm was used to determine the microscopic surface of the films. Images were obtained under constant force, air exposure, and at room temperature. The equipment’s software calculated the average roughness (*R*_a_) based on the arithmetic mean of the absolute surface profile height values.

#### 2.2.6 HEX-3 Release from Films

HEX-3-containing films (1.13 cm^2^) were hydrated in PBS for 5 min and then placed between the donor and receptor compartments of a vertical glass diffusion cell (Franz-type) using a polyester tulle mesh (0.65 cm^2^ pore size) as a support. All films contained 750 ng/cm^2^ of HEX-3. The receptor compartment contained 6 mL of PBS, which was maintained under magnetic stirring (400 rpm) at 25 °C. The films were held in the diffusion cells, and aliquots from the receptor medium were collected at predetermined intervals for up to 72 h. The amount of HEX-3 in the receptor medium was quantified using high-performance liquid chromatography with UV detection (HPLC-UV).

##### 2.2.6.1 HEX-3 Quantification

HEX-3 was quantified using high-performance liquid chromatography with ultraviolet detection (HPLC-UV) (Shimadzu UFLC Prominence, Kyoto, Japan) following a method previous described.^12^ The HPLC system was equipped with a binary pump (LC-20AD), degasser (DGU-20A3 Prominence), autosampler (SIL-20AHT Prominence), and a diode array detector (SPD-M20A Prominence). Chromatographic separations were performed on a reverse-phase Gemini RP-C18 column (250 × 4.6 mm, 3 μm, 110Å) (Phenomenex, CA, USA). Data acquisition and analysis were done using a controller module (CBM-20A Prominence) connected to a computer with Shimadzu LC Solution software. The analytical parameters were as follows: the mobile phase consisted of acetonitrile (A) and water containing 0.1% trifluoroacetic acid (TFA) (B), eluted isocratically in a 10:90 (v/v) ratio over 17 min at a flow rate of 0.5 mL/min. Detection was performed at a wavelength of 210 nm, with the column compartment maintained at 30 °C and an injection volume of 100 μL.

#### 2.2.7 Biocompatibility Studies

##### 2.2.7.1 Skin Cells in a Monolayer Culture

The biocompatibility of films with or without HEX-3 and solutions of HEX-3 at different concentrations was evaluated in human dermal fibroblasts (HDF), HaCaT keratinocytes, human mesenchymal stem cells (hMSCs), and NSC-34 motor neuron cell lines. Cells were seeded in 96-well plates at 2 × 10^4^ cells/well density. After adhesion, they were treated with 100 μL of DMEM containing different concentrations of HEX-3 (100–0.375 μg/mL), and films with or without 750 ng/cm^2^ HEX-3 (2 mm^2^). As a negative control, cells were treated with DMEM 30% DMSO. Following 24 h of incubation, treatments were removed, and 20% resazurin (125 μg/mL) was added to each well for 2 h. Fluorescence was determined using a Synergy Mx microplate reader (BioTek, USA) with excitation at 530 nm and emission at 590 nm. The average percentage of cell viability relative to the positive control was calculated from the obtained values.

##### 2.2.7.2 Hen’s Egg Test-Chorioallantoic Membrane (HET-CAM)

The ocular irritation potential of the films was assessed using the HET-CAM assay. Pathogen-free chicken eggs, weighing between 50 and 75 g, were sourced from a local farm (Pluma Agroavícola, Casa Branca, SP, Brazil). After 10 days of incubation at 37.8 ± 1 °C with 50–70% relative humidity, the eggshell membrane was hydrated and carefully removed to expose the chorioallantoic membrane (CAM). The exposed CAM was treated with films prehydrated in 0.9% NaCl for 5 min. A 1% SDS solution was used as a positive control, and 0.9% NaCl served as a negative control. After 20 s of application, the CAM was gently rinsed with 5 mL of 0.9% NaCl. Irritant effects, such as hemorrhage (bleeding of capillaries and vessels), hyperemia (increased blood flow within vessels, resulting in the appearance of new vessels or intensification of existing ones), and coagulation (interruption of blood flow in vessels, leading to partial or complete disappearance or membrane opacity), were monitored for 5 min using a stereoscopic microscope (SZT – Led, Bel Photonics, Brazil). Each irritant effect was assigned a score based on the time of occurrence^13^ (Supporting Information, Table S2). These scores were summed to calculate the irritation value, which was then used to classify the films as nonirritating (0–0.9), slightly irritating (1–4.9), moderately irritating (5–8.9), or severely irritating (9–21).^13^

#### 2.2.8 Cellular Uptake of HEX-3 Peptide in Monolayer Cultures and Skin Spheroids

##### 2.2.8.1 HEX-3 Peptide—Fluorescein Isothiocyanate (FITC) Conjugation

To conjugate fluorescein isothiocyanate (FITC) to the HEX-3 peptide, which would later be used to evaluate the HEX-3 peptide by fluorescence techniques, a solution of 8.76 mg/mL FITC in DMSO was slowly added dropwise to a 10 mg/mL solution of HEX-3 in PBS 1× under magnetic stirring at 400 rpm. The reaction mixture was allowed to stir for 24 h at 200 rpm. After that, 20 μL of 1 M glycine was added to the solution to react with the excess of FITC, and after 10 min, the mixture was dialyzed using a cellulose acetate membrane (1 kDa) for 24 h to remove any unbound FITC.

The efficiency of FITC conjugation to HEX-3 was determined by a calibration curve using the final product, HEX-3-FITC, solubilized in water and measured using a microplate reader. Dialysis water was analyzed using a Synergy microplate reader to determine the percentage of FITC conjugated to HEX-3 (BioTek, USA). The yield was calculated by subtracting the amount of unbound FITC from the total mass of HEX-3.

##### 2.2.8.2 HEX-3 Cellular Uptake Studies

HEX-3 uptake was assessed in spheroids and monolayer cultures using FITC-conjugated HEX-3. Fluorescence was analyzed by flow cytometry (BD Accuri C6 Plus) and confocal microscopy (Leica SP5). Keratinocytes, dermal fibroblasts, human mesenchymal stem cells, and differentiated motor neurons (HaCaT, HDF, HMSc, and NSC-34, respectively) were individually plated in 24-well plates at a density of 8 × 10^4^ cells/well. A coculture with all was also seeded at a 2 × 10^4^ cells/lineage/well density. Each well received a sterile round coverslip of 13 mm Ø for confocal analysis.

The cells were incubated for 24 h at 37 °C in a 5% CO_2_ atmosphere. After adhesion, the cells were treated with HEX-3-FITC solution (12 μg/mL) in 500 μL of DMEM culture medium under shaking at 50 rpm for 30 min and 4 h. After treatment, for flow cytometry, the cells were trypsinized and centrifuged at 1200 rpm for 5 min, the supernatant was discarded, and the cells were resuspended in 200 μL of culture medium containing 50 μg/mL of Propidium iodide (PI) as a marker for dead cells. All data were analyzed using FlowJo Software (Tree Star, Inc., USA). For the confocal microscope analysis, the cells were fixed with PFA 4% (v/v) for 20 min, washed twice with PBS, and the nuclei were stained with DAPI (500 ng/mL). The slices were mounted using aqueous mounting media, Fluoromount. Images were captured with a 40x oil immersion objective. DAPI was excited with a 405 nm laser and detected in the 409–514 nm range, while FITC (used to label HEX-3) was excited at 488 nm and detected in the 497–554 nm range. Control cells without treatment were used to set parameters and minimize autofluorescence interference.

Regarding skin spheroids, an entire mold (9 × 9) of SS was treated with HEX-3-FITC (50 μg/mL) on day 7° of culture. Alive SS were pipetted (*n* = 3) from the mold, washed twice with PBS, and analyzed by confocal microscope using a z-stack of 6.5 μm in μ-Dish 35 mm (Ibidi). FITC (used to label HEX-3) was excited at 488 nm and detected in the 497–554 nm range and the brightfield. Nontreated SS was used as a control.

#### 2.2.9 HEX-3 Ex Vivo Skin Penetration

##### 2.2.9.1 Ethical Statement

Foreign human skin was obtained after abdominoplastic surgery performed at the Department of Plastic Surgery of Centro Hospitalar Universitário São João (CHUSJ) and Clínica Lotus, Ribeirão Preto, Brazil, after approval from the CHUSJ ethics committee (Protocol 90/17) and Research Ethics Committee (CEP) of the School of Pharmaceutical Sciences of Ribeirão Preto, University of São Paulo (CAAE: 54684521.2.0000.5403), respectively.

##### 2.2.9.2 Ex Vivo Skin Compatibility Assay

Foreign human skin in ages 37 and 69 (both female, abdomen), was cultivated in ex vivo conditions with adaptations^14,15^ to maintain the viability of the tissue, as well as its function intact as a barrier. After tissue preparation, punches of 0.8 cm^2^ were made and positioned inside 12-well inserts. Skin samples were treated for 24 and 48 h with Blank films (without HEX-3), HEX-3-containing films (600 ng) and 15 μL of HEX-3 solution (600 ng). Before treatments, the transepithelial electrical resistance (TEER) was measured and monitored during the treatment regimen. In addition, resazurin was used to measure the tissue viability before, during, and after treatments. Samples of both donors without any treatment were used as controls.

##### 2.2.9.3 Evaluation of Ex Vivo Skin Penetration in Normal Skin and Aged-Mimic Skin

Foreign human skin was cultivated in ex vivo conditions^14,15^ to maintain the tissue’s viability and its function intact as a barrier. Additionally, a protocol to partially remove the SC^15^ was applied to reduce the thickness of epidermis, mimicking the aged skin, which is thinner than younger skin.^16^ After tissue preparation, punches of 0.8 cm^2^ were made and positioned inside 12-well inserts. Skin samples were treated with Blank films, HEX-3-containing films (600 ng), 15 μL of HEX-3-FITC solution (600 ng), and 15 μL of HEX-3 solution (600 ng) for 24 and 48 h. For the 48-h treatment, the films were replaced after 24 h, and an additional 15 μL of HEX-3 solution (with and without FITC) was applied (double dosage). Samples without fluorescence were subjected to histological analysis, H&E staining, and Masson’s Trichrome (TM), to observe tissue architecture with and without each treatment.

Skin and medium samples were collected at multiple time points (0.5, 4, 8, 12, 24, and 48 h) after treatment with HEX-3 solution (50 μg/mL) to analyze penetration kinetics. Additional experimental groups included double treatment (two applications over 24 h with a 12-h interval) and quadruple treatment (four applications over 48 h with 12-h intervals), performed in normal and aged-mimic skin samples to evaluate cumulative absorption. After each time point, the skin sample was frozen in OCT mounting medium for cryo-sectioning, performed in a Cryostat (Epredia) at −20 °C. Sections were obtained at 8 μm and fixed with PFA 4% (v/v). Images were captured with a 20x oil immersion objective. DAPI was excited with a 405 nm laser and detected in the 409–514 nm range, while FITC (used to label HEX-3) was excited at 488 nm and detected in the 497–554 nm range. Control skins without treatment were used to set parameters and minimize autofluorescence interference. Afterward, the media removed from the basolateral compartment were analyzed in a Synergy microplate reader (BioTek, USA) at 490/520 nm.

#### 2.2.10 Efficacy Studies

##### 2.2.10.1 Acetylcholine Release Inhibition

Two different methods were used to evaluate the potential of HEX-3 peptide to inhibit acetylcholine release. Immunofluorescence assessed the expression of the enzyme choline acetyltransferase (ChAT), which is responsible for acetylcholine (ACh) synthesis. Samples treated with a HEX-3 solution (50 μg/mL) for 48 h were incubated with an anticholine acetyltransferase antibody (see Supporting Information for details). Untreated samples served as controls. The samples were analyzed using a Leica TCM SP5 confocal microscope, and ImageJ quantified the fluorescence intensity.

The second method quantified total choline, free choline, and acetylcholine using the Choline/Acetylcholine Quantification Kit from the collected supernatant of cell lysate according to the manufacturer’s instructions. After the cells were differentiated into neurons, they were treated with HEX-3 solution (750 ng/mL) for 48 h.

##### 2.2.10.2 Superoxide Dismutase 2 (SOD2) qRT-PCR Amplification

HaCaT cells were cultured in 24-well plates at a density of 2 × 10^5^ cells/well and incubated for 24 h to adhere. After this period, the cells were treated with films (0.2 cm^2^) containing or not containing 750 ng/cm^2^ of HEX-3 and 50 ng/mL of the HEX-3 solution. Before adding the films to the wells, they were hydrated in 113 μL of PBS. After 48 h of treatments in contact with the cells for the evaluation of superoxide dismutase (SOD2) gene expression, it was determined from the performance of the real-time qPCR. The AllPrep DNA/RNA/Protein Mini Kit was used according to the manufacturer’s recommendations for RNA extraction. First, 350 μL of RLT buffer with β-mercaptoethanol was added to prevent RNA degradation, and the cells were lysed with the help of the rubber policeman (Kasvi, China). The RNA was quantified in the samples using a Nanodrop (Thermofisher, Nanodrop 1000, Massachusetts, USA) with absorbance at 260 nm. The integrity and quality check of the extracted RNA was verified through denaturing agarose gel electrophoresis.

The sample was considered intact when the bands corresponding to the 18S and 28S subunits of rRNA were observed. cDNA (complementary DNA) was synthesized from the intact RNA using the High-Capacity cDNA Reverse Transcription Kits, following the manufacturer’s instructions. In summary, random primers and reverse transcriptase synthesized the complementary single-stranded DNA from the RNA. Nanodrop determined the amount of cDNA, and then all the samples were diluted to a concentration of 50 ng/μL. The qRT-PCR was performed in triplicate using the SYBRgreen method. For the reaction, 5 μL of qPCRBIO SyGreen Blue (PCR Biosystems), 1 μL of RNase-free water, and 1 μL of each primer (forward and reverse) were added, specific to the sequence of the SOD2 gene, which encodes an enzyme present in the mitochondria of cells important in defense against reactive oxygen species. Eight μL of the mix was added to the 96-well plate and 2 μL of cDNA from each sample, diluted in RNase-free water. The plate was sealed and centrifuged at 1500 rpm, at 4 °C for 5 min. The qRT-PCR was performed using the Eppendorf Mastercycler RealPlex2 (Eppendorf, Hamburg, Germany), where the following cycling conditions were programmed: 2 min at 95 °C, followed by 40 cycles of 5 s at 95 °C and 30 s at 60 °C. The data were obtained for subsequent calculation of the relative gene expression. The calculation of the results was performed using the 2^–ΔΔCt^ method. The alpha-ACTN1 gene encodes actin and was used as an endogenous control gene. The sequences of the primers used to amplify the ACTN1 and SOD2 genes can be found in the Supporting Information, Table S3.

##### 2.2.10.3 Detection of Intracellular ROS Neutralizing, Scavenging, and Competition Assay Using CM-H_2_DCFDA

To investigate the capacity of the HEX-3 peptide to neutralize, scavenge, or compete with oxygen reactive species (ROS) in the SS, an assay of ROS induction was performed before, after, and during treatment with Blank films, HEX-3-containing films (600 ng), and 15 μL of HEX-3 solution (600 ng). To teste the neutralization capacity the formulations was incubated 24 h before 250 μM of H_2_O_2_ for 4 h, and the formulations were maintained there during ROS induction; for scavenging 250 μM of H_2_O_2_ was used to induce ROS during 4 h before treatments incubation for 24 h; and for competition study both were added at the same time and incubated for 24 h. After incubation, the SS were washed twice with PBS 1×, then incubated for 30 min with 10 μM of CM-H_2_DCFDA. The fluorescence was measured at 488 nm using a microplate reader.

##### 2.2.10.4 Collagen Type I Increasing Production

Two methods were used to analyze type I collagen production: ELISA and immunofluorescence. SS and human skin explants were treated with Blank film, HEX-3-containing films, and HEX-3 solution at 600 ng for 0.5, 1, 2, 4, 8, 12, 24, and 48 h. When the samples were removed from culture, the skin surface and spheroids were washed to remove the residual treatments, and evaluated regarding collagen I production using ELISA. Briefly, to coat the plate, the rabbit antihuman collagen I primary antibody was diluted to 10 μg/mL in coating buffer, and 100 μL was added to each well of the ELISA plate. The plate was incubated overnight at 4 °C. The plate was washed 3 times with washing buffer. 200 μL of blocking buffer was added to each well, and the plate was incubated for 1 h at room temperature. The plate was washed 3 times with washing buffer, 100 μL of SS lysate or skin homogenate was added to each well, and the plate was incubated for 2 h at room temperature. The standard collagen I curve was prepared in the 0.1–50 ng/mL range. After the samples incubation, the plate was washed 3 times with washing buffer, and wells received 100 μL (1 μg/mL) of primary antibody. 100 μL of the antirabbit HRP-conjugated secondary antibody (1:1000) was added to each well. The plate was incubated for 1 h at room temperature. Finally, the plate was washed 3–5 times with washing buffer, and 50 μL of TMB was added to each well. After 30 min at room temperature in the dark, 50 μL of stop solution was added to each well, and afterward the absorbance was read at 450 nm using a microplate reader. SS lysate and human dermis homogenate was used as a positive control, and PBS as a negative control.

For qualitative and semiquantitative analysis, immunofluorescence staining was performed. Samples treated with a HEX-3 solution (50 μg/mL) for 48 h were incubated with an anticollagen I antibody (see Supporting Information for details). Untreated samples served as controls. The samples were analyzed using a Leica TCM SP5 confocal microscope, and ImageJ was used to quantify the fluorescence intensity.

#### 2.2.11 Statistical Analysis

Student *t* tests were used to compare the means of two populations. For three or more populations, the data were evaluated by analysis of variance (ANOVA), followed by Tukey’s post hoc test, using GraphPad Prism 5.0 software (GraphPad Software, California, USA). Significant values of *p* < 0.05.

## 3 Results and Discussion

### 3.1 Neuronal Differentiation Procedure

Figure 1A–D shows the shapes of neuro-differentiated NSC-34 cells after 4 days of culture with or without RA (1 μM) and Figure 1E–G, neuro-differentiated PC12 cells after 8 days of culture with or without NGF-β (100 ng/mL), along with their corresponding immunofluorescences.

**Figure 1:**
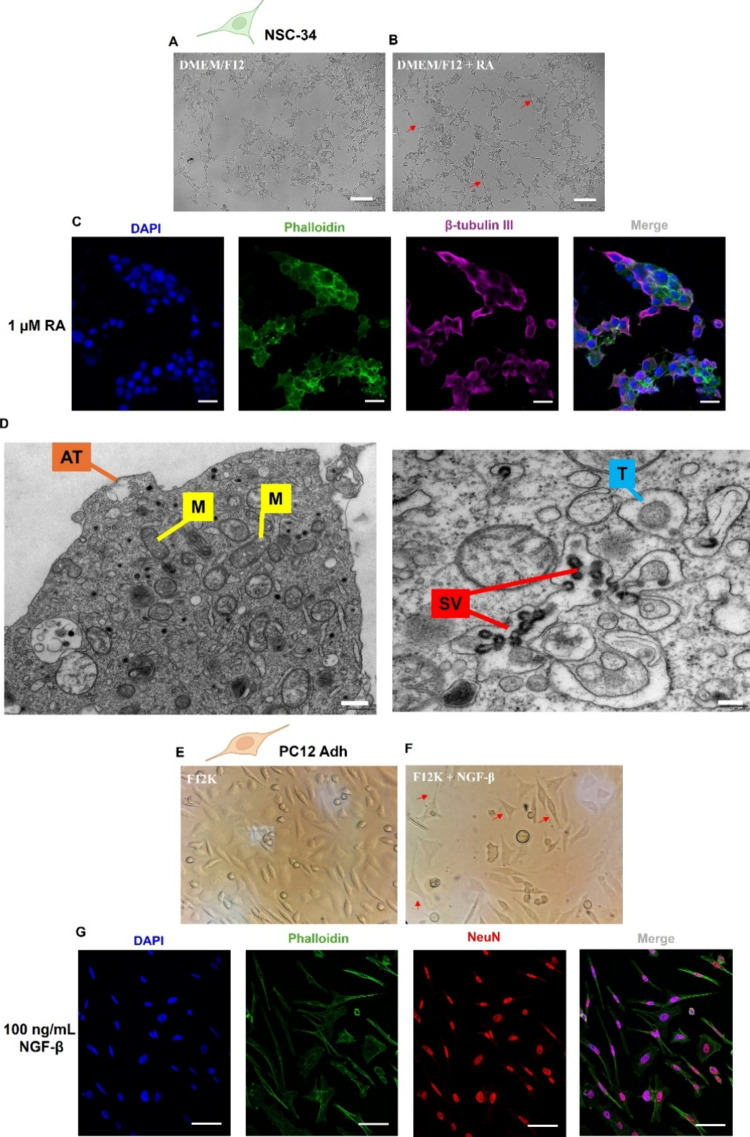
Neurodifferentiation of NSC-34 and PC12 cells. NSC-34 cells were cultured in DMEM/F12 medium supplemented with 1% FBS, 1% penicillin/streptomycin, and 1% MEM-NEAA for 4 days in the (A) absence or (B) presence of 1 μM of RA (Scale bar: 100 μm. The images were obtained using the ZOE Fluorescent Cell Imager). (C) Confocal microscopy of NSC-34 cells treated with RA (20× magnification): the first image shows the fluorescence related to DAPI, the second shows the fluorescence of phalloidin, demonstrating the cytoskeleton of the cells, the third shows the labeling of the antibody β-tubulin III, and the last is the overlay of the images from the blue, green, and violet channels. Scale bar: 20 μm. (D) SEM images of the NSC-34 cells treated with RA. The scale bar indicates 500 and 200 nm for the first and second images, respectively. AT = axon terminal, M = mitochondria, SV = synaptic vesicle, and *T* = terminal (synaptic terminal). (E–G) Neurodifferentiation of PC12 cells after 8 days of culture in F12K medium supplemented with 1% HS and 1% penicillin/streptomycin in the (E) absence or (F) presence of 100 ng/mL of NGF-β. The images were obtained using an inverted microscope with a magnification of 40×. (G) Confocal microscopy of PC12 cells treated with NGF-β (63× magnification). The first image shows the fluorescence related to DAPI, the second shows the fluorescence of phalloidin, the third shows the labeling of the NeuN antibody, and the last is the overlay of the images from the blue, green, and red channels. Scale bar: 50 μm.

Neuronal differentiation is demonstrated by morphological evolution, such as the growth of neurites and their branching, and by functional changes, such as the regulation of neurotransmitters. These processes are influenced by the expression of specific proteins, such as those associated with microtubules.^17^

Figure 1 shows that NSC-34 cells cultured in DMEM/F12, with or without RA (1 μM), exhibit two distinct populations: differentiated neurons with long, branched neurites, and undifferentiated cells with shorter neurites and minimal branching (Figure 1A,B).^18^ However, RA supplementation enhanced and accelerated neuronal differentiation while reducing cell proliferation. To confirm differentiation, β-tubulin III, a neuron-specific cytoskeletal protein, was assessed by immunofluorescence and showed positive staining (Figure 1C).^19^ Additionally, differentiated cells displayed characteristic organelles such as axon terminals, mitochondria, synaptic vesicles, and synaptic terminals (Figure 1D).^20^

PC12 Adh cells, widely used in neurobiology due to their robust differentiation capacity,^21^ also responded to NGF-β (100 ng/mL) after 8 days, showing reduced proliferation, cell body elongation, and neurite outgrowth (Figure 1F). In contrast, unstimulated cells remained elongated but with much shorter neurites (Figure 1E).

Furthermore, upon differentiation with NGF-β (PC12) or RA (NSC-34), both cell types showed increased expression of cholinergic receptors and elevated ChAT and AChE activity.^17,22^ NeuN protein, a marker of mature neurons, was also detected in PC12 cells by immunofluorescence, confirming differentiation (Figure 1G).

### 3.2 Spheroids Development

The skin consists of three layers, epidermis, dermis, and hypodermis, with keratinocytes and fibroblasts being the main cell types in the epidermis and dermis, respectively.^1^ Topical testing commonly uses 2D cell cultures^23,24^ or more advanced 3D coculture models, which better replicate cell–cell and cell–ECM interactions.^25–27^ Although 3D skin spheroid models are established,^6^ there is still a lack of 3D sensory epithelial spheroids for evaluating neuron-targeted therapies. To fill this gap, we developed and characterized two spheroid models: epidermal–dermal spheroids (EDS) and sensory spheroids (SS), designed to evaluate compounds acting on skin structure and neuronal pathways.

#### 3.2.1 EDS Development

Keratinocytes and fibroblasts, the main cell types in the skin,^28^ were chosen as the primary components of the spheroids. To develop the EDS model, cocultures of HaCaT (keratinocytes) and HDF (fibroblasts) cells were prepared in nine different ratios and monitored over 10 days for morphology, diameter, metabolic activity, and histological features (Figure 2).

**Figure 2:**
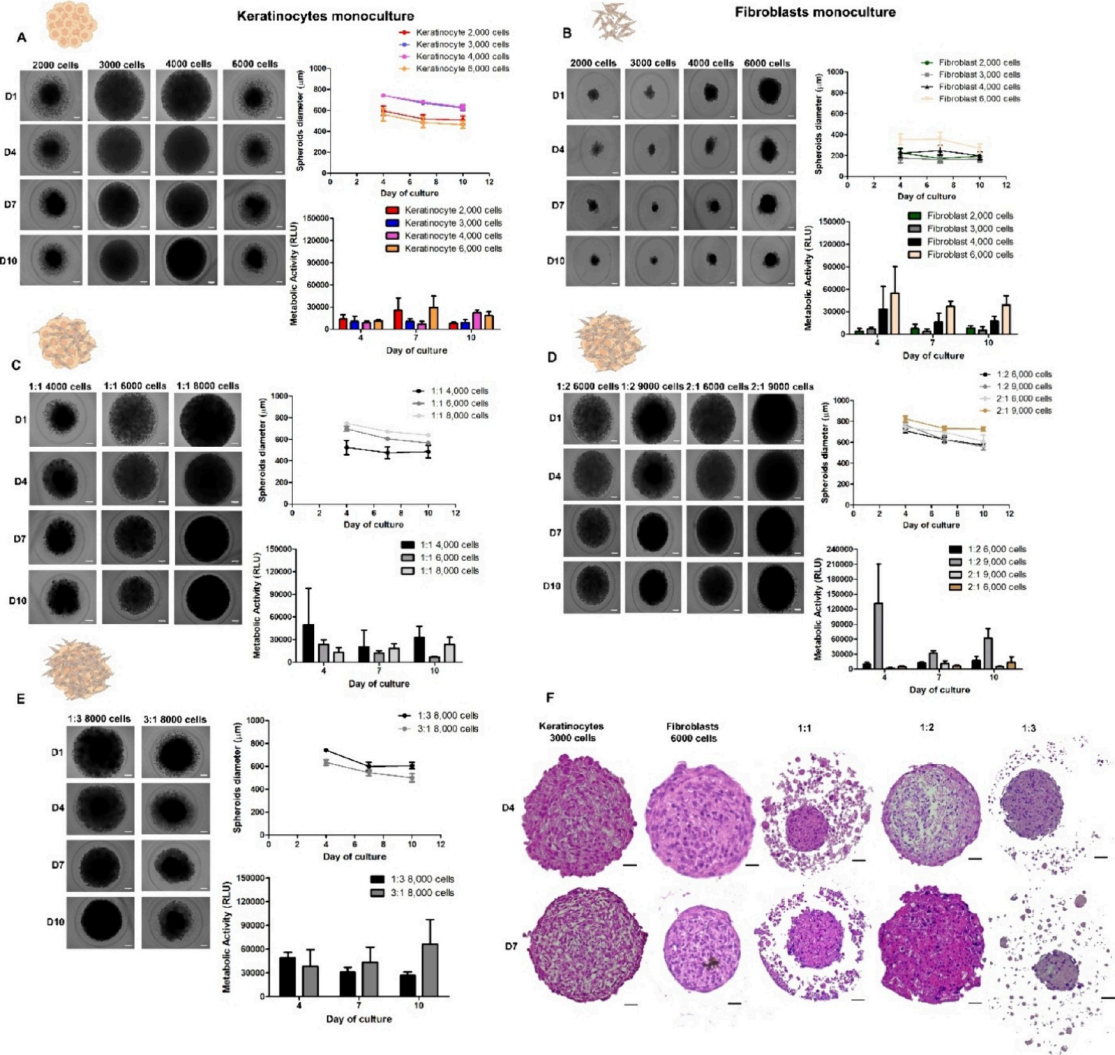
Characterization of EDS formed by monocultures of (A) keratinocytes (HaCaT) or (B) fibroblasts (HDF) and by cocultures of HaCaT: HDF at different ratios: (C) 1:1, (D) 1:2 and 2:1, and (E) 1:3 and 3:1. Bright-field images show spheroid morphology over 10 days (D) in culture (D1, D4, D7, D10). Scale bar: 100 μm. Graphs show spheroid diameter and metabolic activity during the culture period. (F) H&E staining of cocultured spheroids with different HaCaT: HDF ratios at day 10. Scale bar: 50 μm.

Keratinocyte monoculture spheroids displayed larger diameters (623 ± 23 μm) and greater compaction over time compared to the smaller (288 ± 29 μm), irregular fibroblast spheroids, consistent with the lower proliferation and aggregation capacity of fibroblasts.^29^ In cocultures, regardless of the HaCaT: HDF ratio, spheroids became more compact over time, with diameters ranging from 500 to 600 μm by day 10, indicating effective cell–cell interaction and crosstalk.^30^ Additionally, most coculture ratios showed a decline in metabolic activity on days 7 and 10 compared to day 4.

The 1:2 ratio (9000 cells per spheroid) showed the highest metabolic activity on day 4 compared to other conditions. Considering that the epidermis is thinner (60–120 μm) than the dermis (1150–1500 μm),^31^ spheroids with higher fibroblast content, greater total cell number, and favorable features, such as high circularity, compaction, and sustained metabolic activity, were selected for further analysis. These included the 1:1 (8000 cells), 1:2 (9000 cells), and 1:3 (8000 cells) ratios. These conditions proceeded to histological characterization on day 7 (Figure 2F).

Histological analysis of EDS revealed distinct differences in cell organization and ECM production (Figure 2F). The 1:1 ratio (8000 cells) showed fibroblasts mainly at the surface, with notable cell detachment by day 4 and increased detachment, pyknotic nuclei, and vacuoles by day 7, indicating possible cell death and structural instability. In contrast, the 1:2 ratio (9000 cells) exhibited minimal cell detachment at day 4, with fibroblasts concentrated at the periphery and evidence of ECM production. Although initially less compact, these spheroids became denser by day 7, with increased ECM, no necrotic core, and absence of pyknotic nuclei or apoptosis, indicating a more stable and viable model. The 1:3 ratio (8000 cells) had moderate detachment and some pyknotic nuclei by day 4, but by day 7 showed extensive cell loss, disrupted architecture, and apoptotic aggregates.

Given that fibroblasts regulate ECM deposition and cell-matrix adhesion,^32,33^ the 1:2 ratio likely supports better ECM organization and spheroid integrity. Therefore, the HaCaT: HDF ratio of 1:2 (9000 cells per spheroid) was selected for subsequent experiments and designated as the EDS model. Additionally, day 7 of culture was established as the optimal time point for downstream analyses due to high metabolic activity and structural stability.

#### 3.2.2 Quadruple Culture—Sensory Spheroids (SS)

To create a neurosensitive spheroid, the previously selected 1:2 coculture spheroids (9000 cells per spheroid, HaCaT:HDF) were supplemented with 450 neuronal cells (NSC-34) and human mesenchymal stem cells (hMSCs), each representing 5% of the spheroid’s total cell count. This percentage mimics the neuronal component of the skin and the hypodermis, the third and deepest skin layer. The spheroids were cultured for 7 days and assessed for morphology, diameter, metabolic activity, and histology. hMSCs, multipotent progenitor cells capable of differentiating into various mesodermal lineages, such as adipocytes,^34^ were included to represent the hypodermis (Figure 3).

**Figure 3:**
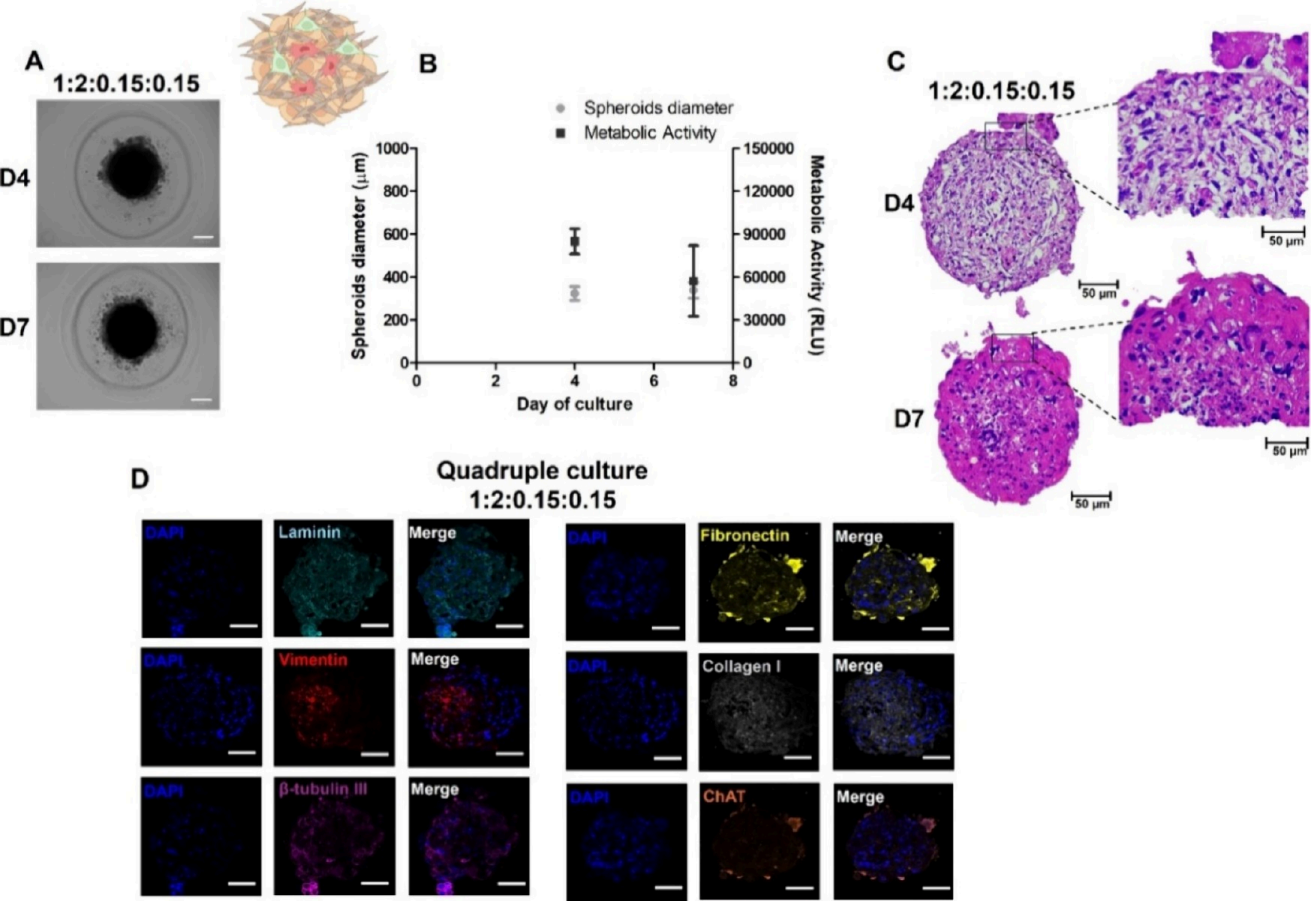
Characterization of SS based on quadruple coculture (HaCaT:HDF:NSC-34:hMSCs). (A) Images of the clear field morphology of the spheroids over 7 days in culture. Scale bar: 100 μm. (B) Diameter of the spheroids over 7 days and metabolic activity of the spheroids in culture. (C) H&E staining of spheroids after 7 days in culture. Scale bar: 50 μm. D4: Day 4 and D7: Day 7 and (D) Immunofluorescence micrographs of representative histological sections. Representative images of the cellular organization of spheroids: Laminin (cyan); Vimentin (red); β-tubulin III (violet); Fibronectin (yellow); Type I collagen (gray) and ChAT (orange) contrasted with DAPI (blue). Scale bar: 50 μm.

As can be seen in Figure 3A, the seeded cells formed a compact structure and spherical SS, similar to the EDS. However, unlike the coculture, they showed small cellular aggregates around the SS, likely corresponding to the remaining NSC-34 maintained over the surface, confirming its presence in the spheroids. The average diameter resulted in half the value of the dual culture (300 ± 38 μm), but with an increase of 15 ± 6 μm between day 4 and day 7, suggesting the cells’ ability to proliferate over the days, forming a compact structure. In general, the outer cells of spheroids measuring 100–300 μm tend to have a greater proliferation potential due to easier access to oxygen and nutrients.^35^ Adding two new cells can modify cell interaction and organization, leading to compaction of the spheroids and a smaller diameter. The metabolic activity profile also decreased over time, demonstrating that adding NSC-34 and hMSCs could not modify this parameter among the analyzed days.

The histological analysis of SS (Figure 3C) revealed reduced cell detachment after 4 days of culture, suggesting early ECM production, later confirmed by immunofluorescence detection of ECM components such as type I collagen, fibronectin, and laminin. After 7 days, the formation of organized cellular aggregates was evident and consistent with morphological observations, indicating that the shed cells did not undergo apoptosis but instead contributed to the structural organization. ECM deposition was prominent, especially on the spheroid surface, while fibroblasts became more concentrated in the inner regions, reinforcing the model’s structural integrity. These findings suggest that SS display improved spheroid stability, reduced peripheral cell loss, and a more homogeneous cellular distribution, indicative of enhanced cell–cell and cell–matrix interactions, and a robust compaction profile.

The distribution and organization of cells in EDS and SS were analyzed by immunofluorescence (IF) (Supporting Information, Figures S1 and 3D, respectively), which confirmed the localization of HaCaT cells, HDF, neurons, and hMSCs and correlated with the results obtained by H&E. The location of the keratinocytes was confirmed by the positive expression of laminin, a protein secreted by the keratinocytes.^36^ Both in the dual culture at a ratio of 1:2 and in the quadruple culture, its presence was evidenced throughout the spheroid, although there was a tendency for it to remain more in the center. The fibroblasts and hMSCs, demonstrated by vimentin staining, a protein present in the filaments of the cytoskeleton of fibroblasts and mesenchymal cells,^37,38^ were observed more inside the spheroids, while in the dual culture, the fibroblasts remained more on the surface, once again demonstrating that the addition of new cells to the spheroids can modify cell distribution, as different types of cells have distinct adhesion and communication properties, causing a cellular reorganization. The large amount of protein deposits in the ECM (type I collagen and fibronectin) is related to the increase in ECM found in histological analyses. Collagen and fibronectin are important proteins for the functioning of the ECM, as collagen provides structural integrity and fibronectin facilitates connections between cells for signal regulation.^39^ Finally, the presence of cholinergic neurons, capable of expressing ChAT enzymes responsible for the synthesis of the neurotransmitter acetylcholine, has been demonstrated through the labeling of the protein present in neuron-specific microtubules, β-tubulin III, and the expression of the ChAT enzyme, respectively. In this way, the neurons were found to be homogeneously distributed throughout the SS and capable of expressing the enzyme ChAT.

Although more advanced functional assays were beyond the scope of this initial characterization study, future investigations could expand the validation of the neuronal component by including electrophysiological recordings to directly assess action potential generation, calcium imaging assays to visualize real-time neuronal activation in response to stimuli, and analysis of neuropeptide release, such as substance P and calcitonin gene-related peptide (CGRP), following stimulation. These approaches would provide a more comprehensive confirmation of sensory neuron functionality and their active participation in the neurocutaneous network modeled in the spheroids.

### 3.3 Antiaging Films Characterization

The polymeric film without HEX-3 was characterized in a previous publication.^9^ Incorporating the HEX-3 peptide, forming HEX3-film, did not significantly change the film’s macroscopic characteristics. Therefore, the films exhibited transparency, a uniform appearance, flexibility, and no points of crystallization or precipitation (Figure 4A). The flexibility and transparency of the films are important factors, as they allow for greater appeal to users when considering their use as an antiaging facial mask and ease of application to the contours of the face.^40^

**Figure 4:**
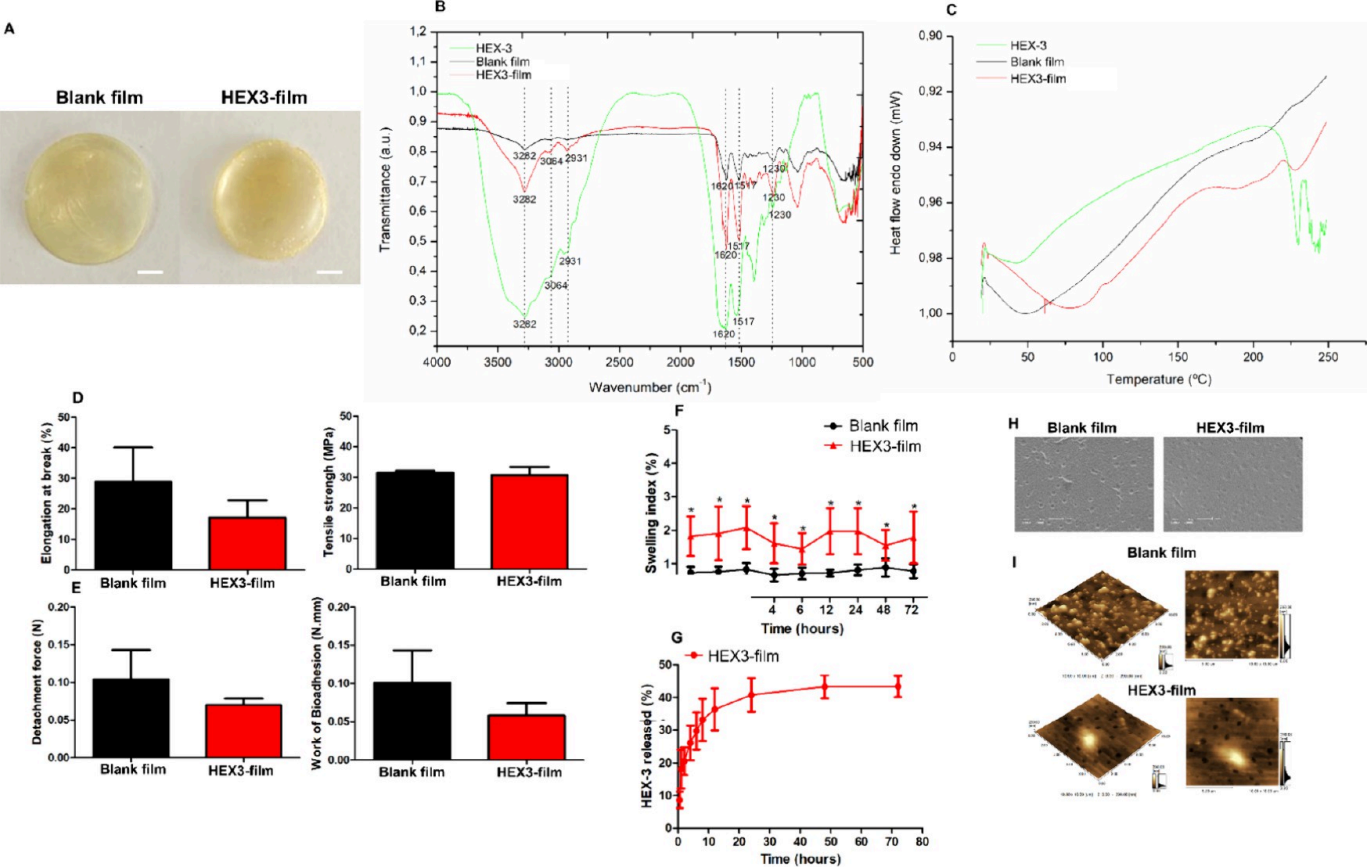
Macroscopic, spectroscopic, thermal, mechanical, and bioadhesive characteristics of films containing or not containing HEX-3. (A) Visual aspect of films. The scale bar indicates 2.5 mm. The images were captured with a 48 MP camera (Apple iPhone 14 ProMax). (B) FTIR spectra of HEX-3 in powder and films, showing absorption between 4000 and 500 cm^–1^. (C) DSC thermograms, with a heating rate of 5 °C/min under continuous nitrogen flow at 3 Kgf/cm^2^. (D) Mechanical properties of films and (E) Bioadhesive properties between the films and human skin at 25 °C. The values are expressed as mean ± standard deviation (SD) (*t* test, *p* ≥ 0.05) (*n* = 5). (F) Swelling index of the films. *Significant difference between the HEX3-film and the blank film at the same time points (*t* test, *p* ≤ 0.05) (*n* = 5). (G) Release profile of HEX-3 from HEX3-film. Linearity was confirmed through an analytical curve of the final HEX-3 conjugate with FITC product over a working concentration range of 109–0.03 μg/mL (*y* = 752.97*x* – 1431.8; *R* ^2^ = 0.9957). Morphological characteristics of the films containing or not HEX-3. (H) SEM images of the films’ surface. The scale bar indicates 2 μm and (I) AFM, tapping mode, of the film without and with HEX-3.

The FTIR analysis had pointed, for HEX-3 peptide powder (Figure 4B), four characteristic peptide bands, at 3282, 1620, 1517, and 1230 cm^–1^, corresponding to the stretching vibrations of amides A, I, II, and III.^41–43^ These regions are generally related to the vibrations of N–H stretching, C=O stretching, angular deformation of NH, and stretching of CN bonds. The broadening and increase in intensity of the peak related to amide A, along with the appearance of bands at 3064 and 2931 cm^–1^,^44^ corresponding to the stretching of amide B, present in the spectrum of the powdered peptide and the HEX3-film, likely indicate the electrostatic interactions between the positive charges of the peptide and the negative charges of silk fibroin and HA.

Thermal analysis was conducted to better understand the interactions of the peptide with the film components and to analyze how the film matrix impacts thermal behavior (Figure 4C). The thermograms of the films without and with HEX-3 showed an endothermic peak at 51 and 80 °C, respectively, corresponding to the evaporation of weakly adsorbed water in the films.^45^ The temperature increase for water evaporation in the HEX3-films suggests that the hydrophilicity of HEX-3 allowed for its adsorption in water, thereby affecting the film’s interface with the water. Furthermore, the presence of the peak at 229 °C, related to the degradation of HEX-3, in the thermograms of the powdered peptide and the HEX3-film confirms an interaction between the peptide and the polymeric matrix.

Mechanical and adhesive properties were evaluated to assess the impact of HEX-3 addition. Although the HEX3-films showed a reduction in elongation break, no significant differences were observed compared to the Blank films. Similarly, tensile strength (Figure 4D), which measures the maximum force that a material can withstand before breaking, and Young’s modulus, which measures the stiffness of the film (1.2 ± 0.5 MPa for HEX3-film vs 1.9 ± 0.5 MPa for Blank film) showed no significant variation.

The interactions between the films and human skin showed that the HEX-3 peptide exhibited a trend in reducing the detachment force and the bioadhesion work (Figure 4E). A lower detachment force indicates that the HEX3-film comes off the skin more easily, in the same way that a lower bioadhesion work measures the energy required to initiate and maintain adhesion, consequently, suggesting a lower effort to its removal from the skin. However, this decrease due to the addition of HEX-3 was insignificant, suggesting that the addition of HEX-3 to the film did not change its mechanical and bioadhesive properties.

The swelling index of the films with HEX-3 increased by about 2 times (1.8 ± 0.6%) compared to the Blank film (0.8 ± 0.2%), maintained throughout the 72-h experiment (Figure 4F). These results corroborate the thermal analyses, which demonstrated that the addition of the hydrophilic peptide could absorb the water present in the film. In general, the addition of the HEX-3 peptide impacts the swelling of the polymeric film, corroborating the thermal and chemical results discussed earlier.

The SEM images show that the addition of HEX-3 did not visually alter the surface of the films (Figure 4H). On the other hand, the AFM images (Figure 4I) revealed changes in topography but not in roughness values (27.69 nm for the Blank film and 26.56 nm for the HEX3-film). We can observe that the interaction of HEX-3 with the polymeric matrix resulted in a somewhat flatter film than that of the Blank films.

HEX-3 was quantified in the receiving solution of the release experiments by HPLC-UV. The calibration curve for HEX-3 in ultrapure water was linear over the concentration range of 2–20 μg/mL (*y* = 92195*x* – 2596.9; *R* ^2^ = 0.9997). Intra- and interday precision demonstrated a coefficient of variation below 4%. The method’s limits of quantification and detection were 0.68 ± 0.014 and 0.22 ± 0.005 μg/mL, respectively. The graph in Figure 4G demonstrates the release profile of HEX-3 from the films. The release of HEX-3 began immediately upon contact with the receiving medium and was sustained throughout the 72 h of the experiment, reaching a cumulative release of 43%. In the first 2 h, 17.55% of the HEX-3 was released, corresponding to 99 μg. The burst release effect probably occurs due to the rapid dissolution of HEX-3 present on the surface of the films. This rapid initial release is particularly relevant for applications such as facial masks, which are designed for overnight use and are sufficient to achieve therapeutic effects. Moreover, the sustained release profile enables broader applications by extending HEX-3 release over time, while the film matrix contributes to enhanced skin hydration and maintenance of active compound stability.^46^

### 3.4 Irritation Potential of the Films and Cytotoxicity

The cell viability results in monocultures of keratinocytes (HaCat), fibroblasts (HDF), motor neuron cell line (NSC-34), and human mesenchymal stem cells (hMSCs) (Supporting Information, Figure S2A–D) showed viability rates of 90.03 ± 5.25, 95.02 ± 3.69, 90.44 ± 6.42, and 91.65 ± 4.38 for Blank film and HEX3-film, respectively, after 24 h of contact. These results indicate that both films were biocompatible under the tested conditions. For the HEX-3 solution, concentrations ranging from 100 μg/mL to 0.375 μg/mL did not significantly reduce cell viability compared to the control group, except at the highest concentration (100 μg/mL) in the hMSC cell line, where a notable 27% decrease in viability was observed. Consequently, a 50 μg/mL concentration of HEX-3 was selected for the subsequent studies.

To further assess biocompatibility, a resazurin assay was performed using human skin samples from both young and elderly donors. The TEER values were consistent with the age of the skin (9841.67 Ω cm^2^ for young skin and 6608.33 Ω cm^2^ for elderly skin), and none of the treatments, Blank film, HEX3-film or 600 ng/mL HEX-3 solution, induced tissue toxicity (Supporting Information, Figure S2E,F). To simulate daily facial film application, the films and HEX-3 solution were reapplied every 12 h (two applications over 24 h and four applications over 48 h). As expected, the negative control (EtOH) significantly reduced skin cell viability by 70 and 64%, validating the assay’s sensitivity. In contrast, all tested treatments, regardless of contact time, skin age, or frequency of application, maintained cell viability above 95% and were statistically similar to the untreated control (*t* test, *p* ≥ 0.05). Thus, the results confirm that the films were noncytotoxic and biocompatible in monolayer cultures and human skin models.

An alternative HET-CAM test was also performed to assess the developed polymeric films’ irritancy potential and confirm their safety (Supporting Information, Table S4). As expected, the 1% SDS solution (positive control) induced vascular damage, while the 0.9% NaCl solution (negative control) caused no visible damage to the chorioallantoic membrane (Supporting Information, Figure S3). Application of the Blank film resulted in hyperemia in one replicate after 29 s, with no changes in the other replicates. The same effect was observed for HEX3-films, with hyperemia in one sample after 3 min and hemorrhage in another after 43 s. Therefore, mild vascular damage was observed in 2 out of 4 HEX3-films, classifying both films as mildly irritating.^13^ These results support the biocompatibility and potential dermocosmetic application of HEX-3-loaded films.

### 3.5 HEX-3 Uptake Studies

To evaluate the cellular uptake of HEX-3, it was first necessary to conjugate the peptide with FITC for tracking purposes. The conjugation efficiency achieved was 87.99 ± 0.36%. The analytical curve of the final HEX-3-FITC was linear across a working concentration range of 109–0.03 μg/mL, as confirmed by the equation *y* = 752.97*x* – 1431.8 (*R* ^2^ = 0.9957).

Figure 5 presents the cellular internalization results of HEX-3, expressed as the percentage of live cells, assessed by flow cytometry after treatment with the HEX-3 solution for 30 min and 4 h. Cells were evaluated both in monoculture and in coculture within the same well. In addition, cocultures were analyzed by confocal microscopy following 4 h of treatment.

**Figure 5:**
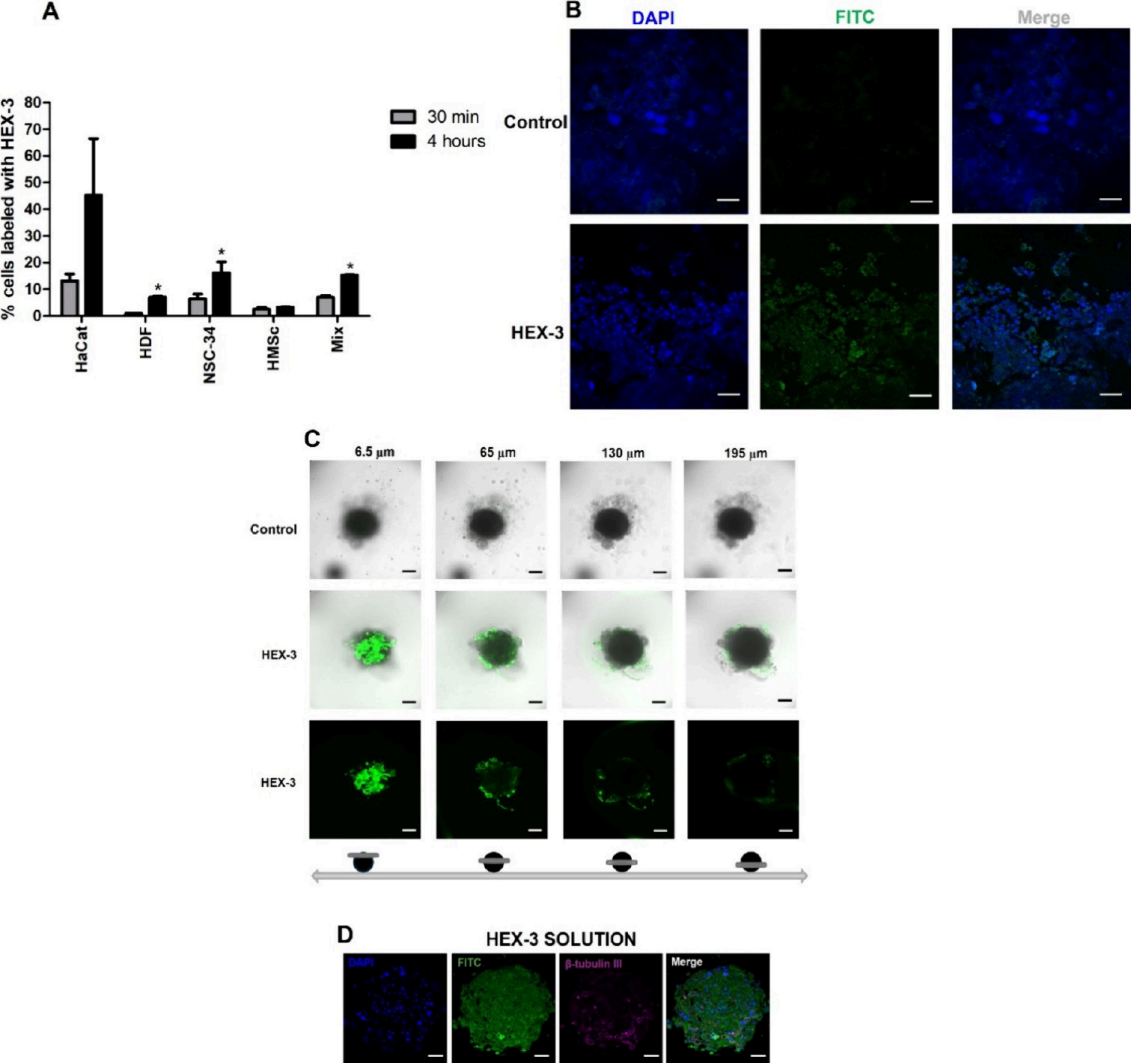
Percentage of live cells that internalized HEX-3 by (A) flow cytometry. *Indicates a significant difference between the 30 min treatment and the 4 h treatment (*t* test, *p* ≤ 0.05, *n* = 2) and (B) Micrographs obtained by confocal microscopy showing the internalization of HEX-3 in the SS (HaCaT, HDF, NSC-34, and hMSCs) after 4 h of treatment with HEX-3 solution. Control: cells treated only with culture medium. Scale bar: 50 μm. (C) Confocal microscopy micrographs showing HEX-3 uptake in SS after 4 h of treatment, obtained at different depths using z-stack optical sectioning. Images include both bright-field and dark-field views at z-positions of 6.5, 65, 130, and 195 μm. FITC fluorescence, indicating HEX-3, was detected in the green channel (λ_exc_ = 488 nm and λ_em_ = 497–554 nm) using 40× magnification. Scale bar: 100 μm. Control: untreated SS incubated with culture medium only. (D) Immunofluorescence micrographs of histological sections from SS generated by quadruple coculture after 48 h of treatment with HEX-3 solution (50 μg/mL). The first quadrant shows DAPI-stained nuclei (blue), the second shows FITC fluorescence corresponding to covalently labeled HEX-3 (green), the third highlights β-tubulin III expression (violet), and the fourth presents the merged image of all channels. Scale bar: 50 μm.

Figure 5A shows that treating individual and mixed cells with the HEX-3 solution for 4 h increased the cellular uptake of HEX-3 compared to the treatment for 30 min. This shows that treatment time is an important factor for the cellular internalization of HEX-3. The uptake of HEX-3 in the cell mix after 4 h was approximately 15%.

The cellular uptake of HEX-3 in the cell mix treated for 4 h was also visualized by confocal microscopy. In Figure 5B, the fluorescence of DAPI in blue identifies the cell nuclei, while the fluorescence of FITC, used as a marker for HEX-3, is visualized in green. The highest intensity of green fluorescence from the FITC, coupled with HEX-3, compared to the control, treated only with culture medium, confirms the cellular internalization of the peptide. Furthermore, it is noted that the internalized HEX-3 is predominantly localized around the cell nucleus, in the cytoplasmic region. However, a concentration of pigments in the cell nucleus can also be observed. It is well-known that the mechanism of cellular uptake depends on the charge and concentration of the peptide and the type of cell.^47,48^ The HEX-3 peptide is positively charged at physiological pH (pI = 7.64) (determined experimentally). The cellular internalization process of cationic peptides seems to be related to the electrostatic interaction with anionic components of the plasma membrane.^49^ The hydrophilic portion of the lipid bilayer contains phosphate and amine groups, which are negatively charged polar moieties that facilitate the interaction with cationic peptides and enhance their cellular uptake. Peptides containing arginine have proven to be the most successful in enhancing cellular internalization through electrostatic interaction with negatively charged membranes.^50^

Considering that 3D models of spheroids can exhibit different behaviors compared to a monolayer culture, the uptake of HEX-3 in SS was also assessed using confocal microscopy following treatment with a HEX-3 solution (50 μg/mL) for 48 h (Figure 5C).

HEX-3 could permeate cells located at various depths within the spheroid, as evidenced by the strong green fluorescence of FITC, used as a marker for HEX-3, compared to the untreated control (Figure 5C). Notably, the highest and most homogeneous uptake occurred in cells at the spheroid surface (6.5 μm) and slightly deeper layers (65 μm), with preferential accumulation around neuronal cells. This was confirmed by the colocalization of FITC fluorescence with β-tubulin III labeling (Figure 5D), a critical finding given that HEX-3 contains a peptide moiety targeting neurotransmitters synthesized by neurons. The higher intensity of FITC fluorescence in the spheroids also suggests that HEX-3 uptake was greater in the 3D coculture model than in the 2D monolayer culture (Figure 5A,B). The 3D culture system offers a more physiologically relevant microenvironment by enabling the synthesis of ECM components and promoting both cell–cell and cell–matrix interactions. Compared to monolayer cultures, cells grown in 3D exhibit distinct surface properties, receptor profiles, metabolic activity, and proliferation rates, leading to outcomes that more closely resemble in vivo conditions.^26^

### 3.6 HEX-3 Ex Vivo Skin Penetration

Although the study using spheroids allows the evaluation of substance uptake by the different cell types that compose the skin, it does not allow the assessment of their penetration through the various layers of the skin. For this purpose, an ex vivo study was conducted using a conventional skin penetration model with human skin.

The process of skin aging causes structural and functional changes in the cells. The epidermis tends to become thinner, with deteriorated keratin fibers and a decreased cell renewal rate and a reduction in water and lipid content, resulting in xerotic skin.^51^ There is also a decrease in the thickness of the dermis, accompanied by a reduction in dermal cells and collagen fibers.^52^ In this way, the behavior of the films containing or not containing HEX-3 on the skin was analyzed by H&E and TM (Figure 6A,B), and the penetration of HEX-3 was examined through confocal microscopy in normal and aged-mimicked skin, following the partial removal of the SC (Figure 6).

**Figure 6:**
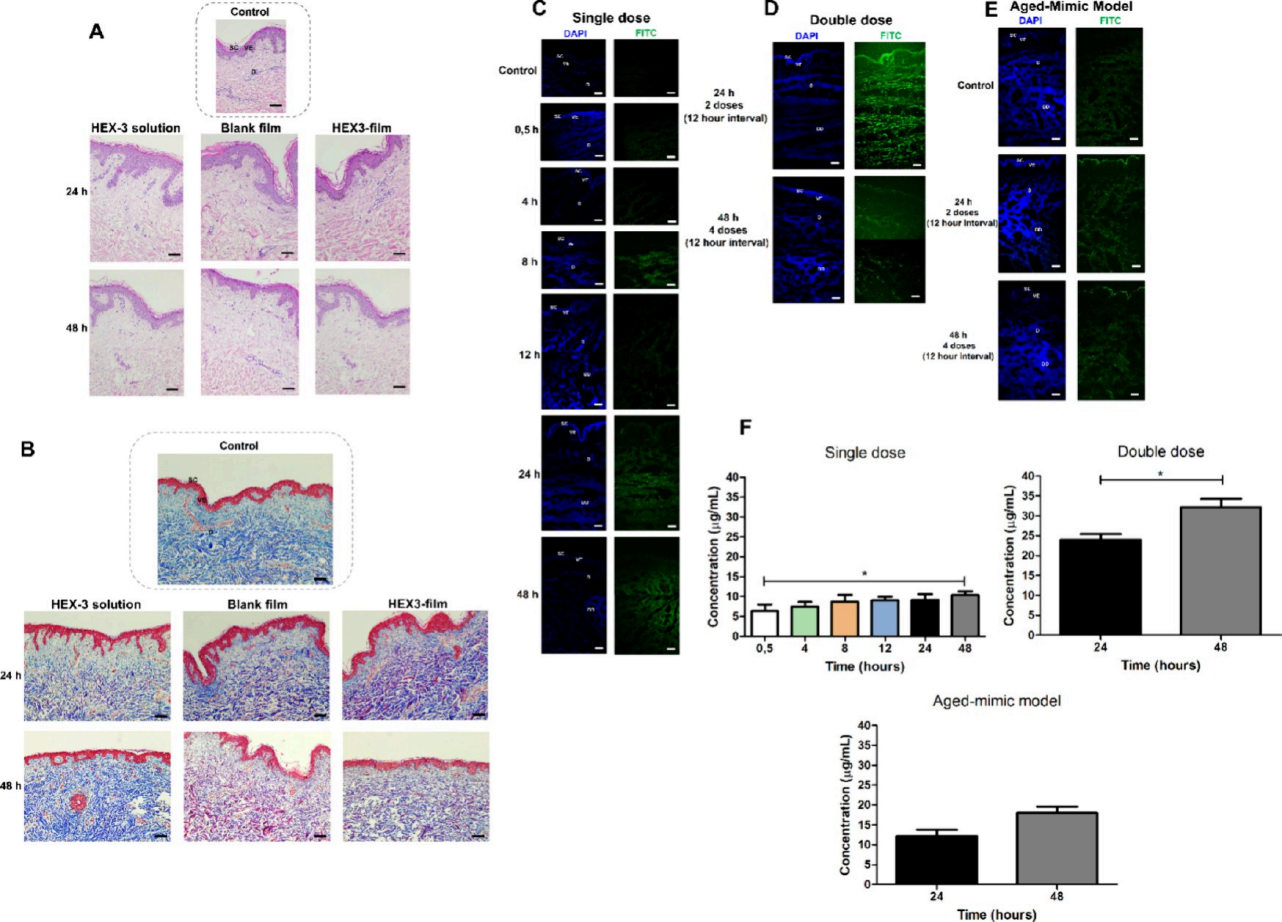
Histological and confocal analysis of human skin treated with HEX-3 solution, blank films, and HEX-3-loaded films for 24 and 48 h. (A) Representative H&E-stained sections and (B) TM-stained sections showing the skin architecture after treatment. SC: stratum corneum; VE: viable epidermis; D: dermis. Control: untreated skin. Scale bars: 100 μm. (C–E) Confocal fluorescence images (*xy* plane) of human skin after treatment with FITC-labeled HEX-3 (50 μg/mL): (C) after a single application for 48 h; (D) untreated normal human skin; (E) aged skin model treated with two doses (24 h) or four doses (48 h). Images acquired in dark-field mode using a confocal microscope (20× magnification, λ_exc_ = 488 nm; λ_em_ = 497–554 nm). FITC fluorescence is shown in green. SC: stratum corneum; VE: viable epidermis; D: dermis; DD: deep dermis. Scale bars: 100 μm. (F) Quantification of HEX-3 in the basolateral compartment. Control: receptor medium from untreated skin. *Indicates significant differences between 0.5 and 48 h, and between 24 and 48 h (ANOVA followed by Tukey’s post hoc test and *t* test, *p* ≤ 0.05, *n* = 4). All fluorescence data were normalized to the control to eliminate background autofluorescence.

The histological examination of the control group revealed normal skin architecture (Figure 6A). The SC displayed the typical arrangement of flattened keratinocytes (corneocytes) filled with keratin. The viable cells of the epidermis exhibited normal characteristics, although some vacuoles were present. Melanin deposits were observed in the epidermis, particularly in the basal layer. The dermis was divided into papillary and reticular layers, consisting of dense and irregular connective tissue with prominent collagen and elastin fibers.

After 24 h of the skin being in contact with the Blank film, the epidermal layer showed an increased hydration profile compared to the control, evidenced by fewer vacuolated epidermal cells and more pronounced fibers within the dermis. However, after 48 h of contact, there were no observable differences compared to the control group. The HEX3-film, after 24 h, showed a more pronounced pattern of hydration and fibers in the dermis than the Blank film. After 48 h, the hydration effect persisted and intensified, with a notable reduction in the scaling of the stratum corneum. The corneocytes were more organized and compact, indicating improved barrier function and structural integrity.

Applying HEX-3 in solution resulted in a histological appearance similar to the control group. However, after 48 h, observable changes occurred in cellular hydration, although these changes were less pronounced than the film treatments. The increase in hydration in the skins treated with films containing HEX-3 is likely related to the presence of the peptide and the polymer HA, one of the polymers present in the polymeric matrix of the films. The HA is a hygroscopic molecule, capable of sequestering a large amount of water,^53^ and for this reason, it contributes to skin hydration.

Maintaining the hydrophilic–lipophilic balance of the skin is extremely important when considering skin aging, as aged skin is also associated with loss of hydration. The hydration of this organ is related to its ability to retain and/or replenish water.^54^ Furthermore, keratins, the main structural proteins of the epidermis, are aggregated and more fibrous, and their filaments break in dry skin exposed to UVB rays. It can be observed that the treatments of the skins with films containing or not containing HEX-3, after 24 and 48 h, were able to increase the production of keratin in an organized manner in both the epidermis and the dermis, as demonstrated by the pink coloration. Meanwhile, the skins treated with HEX-3 solution, after 48 h, showed a collagen quantity similar to the control, marked by the blue coloration (Figure 6B). It is known that skin hydration plays an important role in keratin production; therefore, the HA may have contributed to the increased hydration of the skin surface, allowing for a hydrophilic–lipophilic balance and promoting an improvement in epidermal barrier function and an increase in keratin production.^55^

Figure 6C–E demonstrates the skin penetration of the HEX-3 peptide solution in the different layers of the skin, after 24 and 48 h of treatment, in single or double doses, on normal skin or skin mimicking aging. After 30 min of treatment with a single dose of the HEX-3 solution on normal skin, a slight fluorescence of FITC is already noticeable in the deeper skin layers, indicating the presence of HEX-3. The small size of the peptide molecule facilitates its deeper penetration, as evidenced by the increasing fluorescence intensity observed in the dermis and deeper dermis over time. When the skin was treated with dual doses of the HEX-3 solution, a higher accumulation of HEX-3 in the deeper layers was observed (Figure 6D), compared to the single treatment (Figure 6C), especially at 24 h. In the aging skin model, HEX-3 was detected in the deeper layers and retained in the remaining thin layers of the SC (Figure 6E). The reduction in peptide levels after 48 h (Figure 6C) may be attributed to increased drug permeation through the skin into the basolateral compartment. As a result, the basolateral compartment was quantified using fluorescence measured via a microplate reader (Figure 6F). Regardless of whether the skin had been treated with one or more doses of the HEX-3 solution, the peptide was able to permeate all layers of the skin, reaching the basolateral compartment. When the skin was treated with a dose of the HEX-3 solution, approximately 10 μg/mL (corresponding to 20% of the applied dose) reached the basolateral compartment after 48 h. While normal skin received a double dose, the concentration of HEX-3 in the basolateral compartment was three times higher (30 μg/mL), unlike the aged-mimicked skins (18 μg/mL), since the skin exhibited cellular disorganization, hindering the effective penetration of the substances. Furthermore, the decrease of the peptide inside the skin layers after 48 h of treatment (Figure 6D) is related to its greater penetration through the skin, reaching the basolateral compartment.

These findings confirm the ability of HEX-3 to penetrate and distribute across different skin layers and provide indirect evidence of its stability during the permeation process. Detecting intact HEX-3, labeled with FITC, in the deeper layers after 24 and 48 h suggests that the peptide resists degradation while traversing the skin barrier. Although the current study did not include dedicated stability assays under conditions mimicking the skin surface, such as acidic pH and exposure to serine proteases, the preserved fluorescence signal and peptide accumulation in deeper layers indicate that HEX-3 maintains structural integrity upon topical application. Future studies are warranted to systematically evaluate HEX-3 stability under simulated cutaneous conditions to characterize its degradation profile and fully optimize formulation strategies.

Effective skin penetration of the neuropeptide alone is insufficient to ensure its biological activity. Therefore, studies using the SS model are essential to assess its ability to inhibit acetylcholine release and other potential effects once inside the skin, as presented and discussed below.

### 3.7 Efficacy Study

Acetylcholine (ACh) is a neurotransmitter synthesized by the enzyme choline acetyltransferase (ChAT) in cholinergic neuronal cells such as PC12 and NSC-34. At neuromuscular junctions and synapses, ACh is hydrolyzed by acetylcholinesterase (AChE) into choline and acetate.^56^ This neurotransmitter plays a key role in several physiological processes, including attention, memory, and muscle contraction.^57^

Figure 7 shows that the fluorescence intensities of β-tubulin III and ChAT were lower in the SS treated with the HEX-3 solution (3.85 and 18.42, respectively) for 48 h compared to the untreated control (9.93 and 22.40, respectively). The synthesis rate of ACh is primarily limited by choline uptake from the synaptic cleft into the presynaptic terminal. However, ChAT activity can also be regulated by membrane depolarization and Ca^2+^ influx, which trigger ACh release and subsequently increase choline availability at nerve terminals.^58^ HEX-3, which has an N-terminal sequence identical to the SNAP-25 protein, can compete with SNAP-25 for incorporation into the SNARE complex, destabilizing it and consequently reducing muscle contractions.^59^ Based on these findings, it is suggested that treatment with HEX-3 for 48 h inhibited ACh release, as indicated by the reduced fluorescence signal of ChAT. Furthermore, HEX-3 may have bound to SNARE complex receptors, interfering with the binding of the β-tubulin III antibody and thereby reducing its fluorescence signal as well.

**Figure 7:**
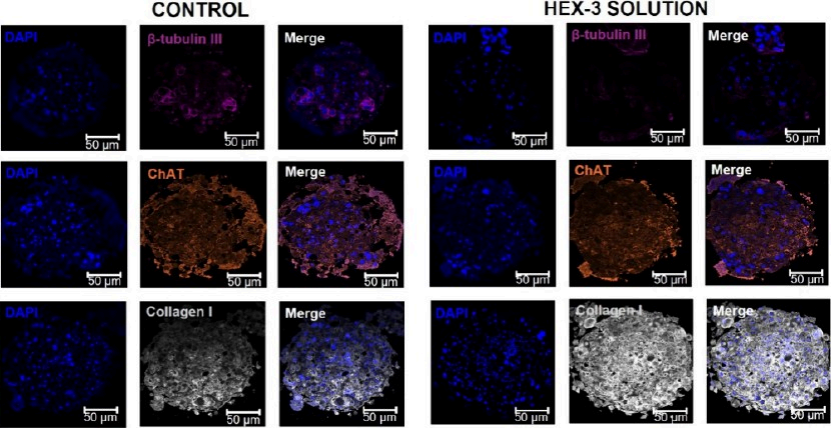
Immunofluorescence micrographs of representative histological sections of SS based on quadruple coculture. Representative images of the cellular organization of the spheroids after treatment with HEX-3 solution (50 μg/mL) for 48 h: β-tubulin III (violet), ChAT (orange), and type I collagen (gray) contrasted with DAPI (blue). Scale bar: 50 μm. Control: untreated spheroids.

Complementary results with PC12 Adh cells and another ACh quantification method are shown in Figure 8A, which suggests an increase in ACh within the PC12 cells, thus implying that the amount of ACh released into the synaptic clefts was lower after treating the cells with the HEX-3 solution.

**Figure 8:**
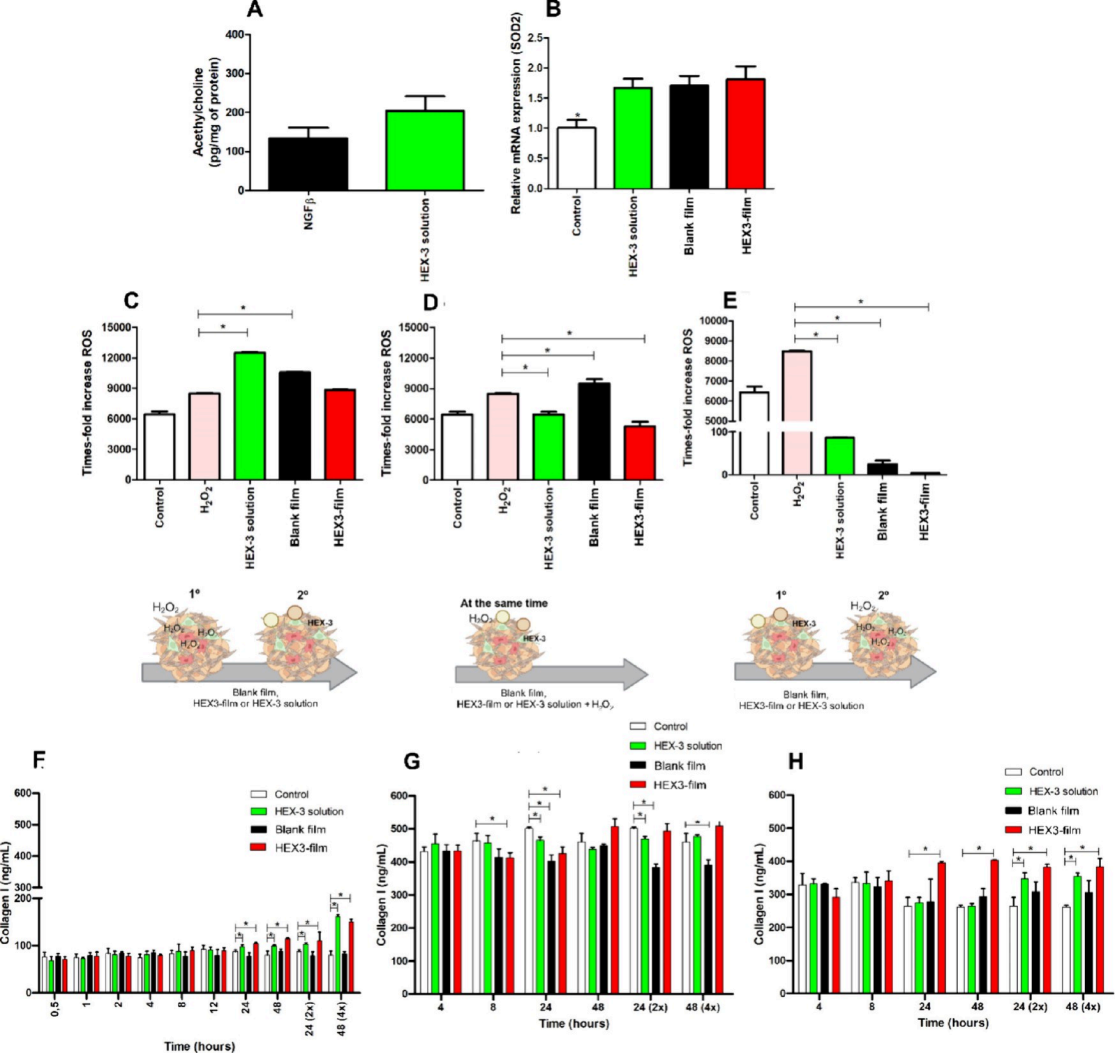
(A) Effect of the HEX-3 solution (750 ng/mL) on ACh synthesis in PC12 Adh cells after 48 h of treatment, compared to the NGF-β (100 ng/mL) control group (*t* test, *p* ≥ 0.05, *n* = 3). (B) Relative mRNA expression of SOD2 in HaCaT cells after 48 h of treatment with the HEX-3 solution (50 ng/cm^2^), Blank film, or HEX-3-loaded film. *Indicates a statistically significant difference compared to the untreated control (*t* test, *p* ≤ 0.05, *n* = 3). (C–E) Intracellular ROS levels in SS after treatment with HEX-3 solution, Blank film, or HEX-3-loaded film, assessed using the CM-H2DCFDA fluorescent probe: (C) ROS scavenging, (D) competition with ROS generation, (E) ROS neutralization. Control: cells treated with a culture medium only. *Indicates a statistically significant difference compared to the H_2_O_2_-treated group (*t* test, *p* ≤ 0.05, *n* = 3). (F–H) Effect of the HEX-3 solution, Blank film, and HEX3-film on collagen production, as measured by ELISA, in (F) SS, (G) young human skin, and (H) elderly human skin at different time points. *Indicates a statistically significant difference compared to the control group at the corresponding time point (*t* test, *p* ≤ 0.05, *n* = 3).

Through the qRT-PCR, it was possible to evaluate the gene expression of the enzyme superoxide dismutase 2 (SOD2), an enzyme located in the mitochondrial matrix of cells and representing the first line of antioxidant defense. It converts the superoxide anion into hydrogen peroxide and molecular oxygen, which can then be eliminated. In aged senescent skin, increased concentrations of ROS, as well as an imbalance in the expression and regulation of SOD2 mRNA, are demonstrated.^60^ A significant positive regulation of SOD2 in HaCaT cells treated with HEX-3 solution, Blank films, and HEX3-films was observed in Figure 8B. Thus, the positive regulation of the enzyme SOD2 confirms a positive effect of the HEX-3 peptide and the polymeric films in protecting against oxidative stress. The antioxidant effect of the films can also be attributed to the presence of chitosan,^61^ one of the polymers found in the polymeric film matrix.

Stressful conditions in cells, such as exposure to ultraviolet radiation, can elevate intracellular ROS levels, contributing to oxidative stress and subsequent cellular damage.^62^ The potential of the HEX-3 solution and films with or without HEX-3 to scavenge, compete with, or neutralize intracellular ROS was evaluated in SS using the CM-H_2_DCFDA fluorescent probe (Figure 8C–E). After entering the cells, the acetate groups of CM-H_2_DCFDA are cleaved by intracellular esterases, producing the nonfluorescent compound DCFH. In the presence of ROS, DCFH is oxidized to dichlorofluorescein (DCF), a highly fluorescent compound, which can be quantified using fluorescence-based assays.^63^

The results showed that treatments with the HEX-3 solution, Blank film, and HEX-3-loaded film could not scavenge ROS when applied after oxidative stress was induced by H_2_O_2_ (Figure 8C). However, when the HEX-3 solution and HEX-3-containing films were administered simultaneously with (Figure 8D) or before (Figure 8E) the oxidative stress inducer, a significant reduction in ROS levels was observed compared to cells treated with H_2_O_2_ alone, including those treated with the Blank film.

Antioxidant agents typically neutralize ROS by directly reacting with them, thus halting chain reactions and protecting cells from oxidative damage.^64^ These findings suggest that both the HEX-3 solution and the films, regardless of whether they contained HEX-3, exhibited antioxidant activity by preventing ROS generation. For the Blank film, this activity is likely attributable to HA, as supported by previous studies.^65,66^ These observations are consistent with the upregulation of SOD2 expression observed under the same conditions.

Type I collagen fibers account for approximately 75% of the total collagen content in young skin; however, with aging, this proportion decreases to about 25%.^67^ These fibers are critical in maintaining the skin’s mechanical properties.^68^ Treatment of SS with the HEX-3 solution resulted in a type I collagen fluorescence intensity 2.3 times higher than that of the control group (Figure 7), indicating enhanced collagen production. These findings are consistent with previous in vivo studies^69^ and are further supported by the results obtained using the ELISA method (Figure 8F–H).

The ELISA-based quantification of type I collagen provides data comparable to literature reports.^67^ In the control group, collagen levels in aged skin were approximately 300 ng/mL, while in young skin, they reached around 450 ng/mL. The decline in types I and III collagen observed in aged skin is attributed to decreased fibroblast-mediated collagen synthesis and increased degradation by matrix metalloproteinases (MMPs).^70^

In young skin samples (Figure 8G), treatment with the HEX-3 solution and HEX-3-loaded films did not lead to statistically significant increases in collagen levels; however, a trend toward increased production was observed after 48 h of exposure. In contrast, aged skin (Figure 8H), which naturally contains lower levels of type I collagen, showed a significant increase in collagen production following treatment with either one or two applications of the HEX-3 solution or HEX3-film. The Blank film without HEX-3, however, did not produce any effect.

The SS models developed in this study produced results that corroborate previous immunofluorescence findings (Figure 7), confirming the presence of type I collagen within these models. Notably, although baseline collagen production in untreated SS was lower than in young and aged human skin, treatment with the peptide, whether in solution or film form, led to collagen levels comparable to those in aged skin (Figure 8H).

These findings validate the SS models as a reliable platform for assessing the biological effects of topically applied HEX-3 and suggest that they closely mimic aged skin, making them a valuable tool for screening compounds that influence collagen synthesis. However, a limitation of the current model is the absence of immune cell types, which restricts its ability to fully recapitulate the complex neuro-immune interactions involved in skin homeostasis and inflammation. For future refinement, incorporating monocyte-derived macrophages, which could be activated by inflammatory stimuli such as lipopolysaccharide, would enable the simulation of immune responses and expand the model’s applicability to study conditions like sensitive skin, dermatitis, and inflammatory aging.

## 4 Conclusions

In summary, this study successfully established a novel sensory neuron-integrated skin spheroid (SS) model that incorporates key skin cell types, keratinocytes, fibroblasts, adipocytes, and sensory neurons, providing a physiologically relevant platform for evaluating neuropeptide-based topical delivery systems. Our findings demonstrate that the SS model effectively internalizes the neuropeptide HEX-3, particularly around neuronal cells, validating its specificity and biological relevance. While ex vivo skin penetration studies confirmed that HEX-3 can traverse the SC and accumulate in deeper layers, the SS model uniquely enabled the assessment of HEX-3′s biological activity, including acetylcholine release inhibition, antioxidant defense enhancement via SOD2 upregulation, and stimulation of type I collagen synthesis. Notably, treatment with HEX-3 in solution and polymeric film forms improved the skin’s structural integrity and hydration while mitigating oxidative stress when applied preventively. Moreover, HEX-3 significantly boosted collagen production in aged skin samples, an effect mirrored in the SS model, further validating its utility as a screening tool for antiaging and skin-repair therapies. Overall, this integrated approach underscores the potential of the SS model as a valuable platform for preclinical evaluation of topical neuropeptide delivery systems and their biological effects on skin.

## Supplementary Material



## Notes

List of antibodies and staining used for immunofluorescence imaging of SS and EDS; scores attributed to the observed effects on the chorioallantoic membrane as a function of time after treatment with the films; primers used in qRT-PCR amplification; classification of the effects of films with and without HEX-3 in the HET-CAM assay after 5 min of contact, according to (Luepke; Kemper, 1986), Sodium Lauryl Sulfate 1% (SDS) was the positive control, while Sodium Chloride 0.9% (NaCl) was the negative control, values expressed as mean ± standard deviation (SD) (*n* = 4); immunofluorescence micrographs of representative histological sections of SS spheroids in double culture at a ratio of 1:2 (HaCaT:HDF); representative images of the cellular organization of spheroids: Laminin (cyan); Vimentin (red); β-tubulin III (violet); Fibronectin (yellow); Type I collagen (gray) and ChAT (orange) contrasted with DAPI (blue), scale bar: 50 μm; cell viability of monoculture cells (A) keratinocytes (HaCaT), (B) fibroblasts (HDF), (C) neurons-like (NSC-34), and (D) human mesenchymal stem cells (hMSCs) after treatment with the film containing or not HEX-3 and different concentrations of HEX-3 solution (100–0.375 μg/mL) (*t* test, *p* ≤ 0.05 (*n* = 5), *significant difference between control and treatment, cell viability of (E) young and (F) elderly skin treated with the film containing or not HEX-3 and HEX-3 solution (600 ng), EtOH 80% was used negative control (*t* test, *p* ≤ 0.05, *n* = 3), *significant difference between control and EtOH; and vascular effects on the chorioallantoic membrane in the HET-CAM assay monitored for 5 min after treatment with the films, 1% SDS: Positive control and 0.9% NaCl: Negative control, black arrow: hemorrhage (PDF)

## Abbreviations

PBS: phosphate buffer saline
ChAT: acetyltransferase
HEX-3: acetylhexapeptide-3
SS: sensory spheroids
3D: three-dimensional
2D: two-dimensional
hMSCs: human mesenchymal stem cells
MMPs: metalloproteinase enzymes
DCF: dichlorofluorescein DCF
ROS: oxygen reactive species
SOD2: enzyme superoxide dismutase 2
H_2_O_2_: hydrogen peroxide
SDS: sodium lauryl sulfate
EtOH: ethanol
NaCl: sodium chloride
HA: hyaluronic acid
RA: retinoic acid
SD: standard desviation
HDF: human dermis fibroblast
IF: immunofluorescence
ECM: extracellular matrix
FBS: fetal bovine serum
MEM-NEAA: nonessential amino acids
EDS: epidermal–dermal spheroid
FITC: fluorescein isothiocyanate
DMSO: dimethyl sulfoxide
HS: horse serum
RT: room temperature
AChE: enzyme acetylcholinesterase
H&E: hematoxylin and eosin
TM: masson trichrome
SEM: scanning electron microscopy
AFM: atomic force microscopy
ACh: acetylcholine
DMEM: Dulbecco’s Modified Eagle Medium
TFA: trifluoroacetic acid
DSC: differential scanning calorimetry
FTIR: Fourier transform infrared spectroscopy
ATR: attenuated total reflectance
HPLC-UV: high-performance liquid chromatography with ultraviolet detection
RT: relative humidity
PFA: paraformaldehyde
NGF-β: Neural factor growth
HET-CAM: Hen’s egg test-chorioallantoic membrane
qRT-PCR: real-time qPCR
PI: propidium iodide
TEER: transepithelial electrical resistance
CAM: chorioallantoic membrane
NP: nanoparticle
PVA: poly­(vinyl alcohol)

## References

[1] Lai-Cheong J. E., McGrath J. A. (2021). Structure and Function of Skin. Hair and Nails. Medicine.

[2] Pegoraro N., Gehrcke M., Camponogara C., Fialho M., Cruz L., Oliveira S. (2024). The Association of
Oleic Acid and Dexamethasone Acetate
into Nanocapsules Enables a Reduction in the Effective Corticosteroid
Dose in a UVB Radiation-Induced Sunburn Model in Mice. Pharmaceutics.

[3] Brighenti M. de S., Montanheri L. R. da S., Duque M. D., Andreo-Filho N., Lopes P. S., Garcia M. T. J., Mackenzie L., Leite-Silva V. R. (2025). *In Vitro* Drug Release and *Ex Vivo* Dermal Drug Permeation Studies of Selected Commercial
Benzoyl Peroxide Topical Formulations: Correlation Between Human and
Porcine Skin Models. Mol. Pharmaceutics.

[4] Zoio P., Ventura S., Leite M., Oliva A. (2021). Pigmented Full-Thickness
Human Skin Model Based on a Fibroblast-Derived Matrix for Long-Term
Studies. Tissue Eng. Part C Methods.

[5] Kim B. S., Ahn M., Cho W.-W., Gao G., Jang J., Cho D.-W. (2021). Engineering
of Diseased Human Skin Equivalent Using 3D Cell Printing for Representing
Pathophysiological Hallmarks of Type 2 Diabetes in Vitro. Biomaterials.

[6] Granato G., Ruocco M. R., Iaccarino A., Masone S., Calì G., Avagliano A., Russo V., Bellevicine C., Di Spigna G., Fiume G., Montagnani S., Arcucci A. (2017). Generation and Analysis
of Spheroids from Human Primary
Skin Myofibroblasts: An Experimental System to Study Myofibroblasts
Deactivation. Cell Death Discovery.

[7] Choi E. H. (2019). Aging of
the Skin Barrier. Clin Dermatol.

[8] Lungu C., Considine E., Zahir S., Ponsati B., Arrastia S., Hallett M. (2013). Pilot Study
of Topical Acetyl Hexapeptide-8 in the
Treatment for Blepharospasm in Patients Receiving Botulinum Toxin
Therapy. Eur. J. Neurol.

[9] Martin B. A., Dalmolin L. F., Lemos C. N., de Menezes Vaidergorn M., da Silva Emery F., Vargas-Rechia C. G., Ramos A. P., Lopez R. F. V. (2024). Electrostimulable
Polymeric Films with Hyaluronic Acid and Lipid Nanoparticles for Simultaneous
Topical Delivery of Macromolecules and Lipophilic Drugs. Drug Deliv Transl Res..

[10] Lemos C. N., Cubayachi C., Dias K., Mendonça J. N., Lopes N. P., Furtado N. A. J. C., Lopez R. F. V. (2018). Iontophoresis-Stimulated
Silk Fibroin Films as a Peptide Delivery System for Wound Healing. Eur. J. Pharm. Biopharm..

[11] Pawar H. V., Tetteh J., Boateng J. S. (2013). Preparation,
Optimisation and Characterisation
of Novel Wound Healing Film Dressings Loaded with Streptomycin and
Diclofenac. Colloids Surf. B Biointerfaces.

[12] Lim S. H., Sun Y., Thiruvallur
Madanagopal T., Rosa V., Kang L. (2018). Enhanced Skin
Permeation of Anti-Wrinkle Peptides via Molecular Modification. Sci. Rep.

[13] Luepke N. P., Kemper F. H. (1986). The HET-CAM test: an alternative to the
draize eye test.

[14] Leite M. N., Viegas J. S. R., Praça F. S. G., de Paula N. A., Ramalho L. N. Z., Bentley M. V. L. B., Frade M. A. C. (2021). Ex Vivo Model
of Human Skin (HOSEC) for Assessing the Dermatokinetics of the Anti-Melanoma
Drug Dacarbazine. European Journal of Pharmaceutical
Sciences.

[15] Viegas J., Dias S., Carvalho A. M., Sarmento B. (2023). Characterization of
a Human Lesioned-Skin Model to Assess the Influence of Skin Integrity
on Drug Permeability. Biomedicine & Pharmacotherapy.

[16] Farage M. A., Miller K. W., Elsner P., Maibach H. I. (2013). Characteristics
of the Aging Skin. Adv. Wound Care (New Rochelle).

[17] Maier O., Böhm J., Dahm M., Brück S., Beyer C., Johann S. (2013). Differentiated NSC-34 Motoneuron-like
Cells as Experimental Model for Cholinergic Neurodegeneration. Neurochem. Int..

[18] Bandla A. C., Sheth A. S., Zarate S. M., Uskamalla S., Hager E. C., Villarreal V. A., González-García M., Ballestero R. P. (2023). Enhancing
Structural Plasticity of PC12 Neurons during
Differentiation and Neurite Regeneration with a Catalytically Inactive
Mutant Version of the ZRICH Protein. BMC Neurosci..

[19] Flynn K. C. (2013). Cytoskeleton and
Neurite Initiation. Bioarchitecture..

[20] Schikorski T., Stevens C. F. (2001). Morphological Correlates of Functionally Defined Synaptic
Vesicle Populations. Nat. Neurosci.

[21] Chua P. F., Lim W. K. (2021). Optimisation of
a PC12 Cell-Based in Vitro Stroke Model
for Screening Neuroprotective Agents. Sci. Rep..

[22] Greene, L. A. ; Rukenstein, A. Issue Ai Dune 25; 1981; Vol. 256.

[23] Grosicki, M. ; Latacz, G. ; Szopa, A. ; Cukier, A. ; Kieć-Kononowicz, K. The Study of Cellular Cytotoxicity of Argireline®-an Anti-Aging Peptide*. www.actabp.pl. 24644551

[24] López-García J., Lehocký M., Humpolíček P., Sáha P. (2014). HaCaT Keratinocytes
Response on Antimicrobial Atelocollagen Substrates: Extent of Cytotoxicity,
Cell Viability and Proliferation. J. Funct Biomater.

[25] Klicks J., Maßlo C., Kluth A., Rudolf R., Hafner M. (2019). A Novel Spheroid-Based
Co-Culture Model Mimics Loss of Keratinocyte Differentiation, Melanoma
Cell Invasion, and Drug-Induced Selection of ABCB5-Expressing Cells. BMC Cancer.

[26] Ravi M., Paramesh V., Kaviya S. R., Anuradha E., Solomon F. D. P. (2015). 3D Cell
Culture Systems: Advantages and Applications. J. Cell Physiol.

[27] Viegas J., Costa S., Dias S., Pereira C. L., Sarmento B. (2024). Patient-Derived
Melanoma Immune-Tumoroids as a Platform for Precise High Throughput
Drug Screening. Adv. Sci..

[28] Baroli B. (2010). Penetration
of Nanoparticles and Nanomaterials in the Skin: Fiction or Reality?. J. Pharm. Sci..

[29] Russo B., Brembilla N. C., Chizzolini C. (2020). Interplay Between Keratinocytes and
Fibroblasts: A Systematic Review Providing a New Angle for Understanding
Skin Fibrotic Disorders. Front. Immunol..

[30] Sargenti A., Musmeci F., Bacchi F., Delprete C., Cristaldi D. A., Cannas F., Bonetti S., Pasqua S., Gazzola D., Costa D., Villa F., Zocchi M. R., Poggi A. (2020). Physical Characterization
of Colorectal Cancer Spheroids and Evaluation of NK Cell Infiltration
Through a Flow-Based Analysis. Front. Immunol..

[31] Oltulu P., Tekecik M., Taflioglu
Tekecik Z., Kilinc F., Ince B. (2022). Measurement
of Epidermis, Dermis, and Total Skin Thicknesses from Six Different
Face Regions. Selcuk Tip Dergisi.

[32] Cole M. A., Quan T., Voorhees J. J., Fisher G. J. (2018). Extracellular Matrix
Regulation of Fibroblast Function: Redefining Our Perspective on Skin
Aging. J. Cell Commun. Signal..

[33] Tan Y., Suarez A., Garza M., Khan A. A., Elisseeff J., Coon D. (2020). Human Fibroblast-Macrophage
Tissue Spheroids Demonstrate Ratio-Dependent
Fibrotic Activity for *in Vitro* Fibrogenesis Model
Development. Biomater Sci..

[34] Chen Q., Shou P., Zheng C., Jiang M., Cao G., Yang Q., Cao J., Xie N., Velletri T., Zhang X., Xu C., Zhang L., Yang H., Hou J., Wang Y., Shi Y. (2016). Fate Decision
of Mesenchymal Stem
Cells: Adipocytes or Osteoblasts?. Cell Death
Differ..

[35] Friedrich J., Ebner R., Kunz-Schughart L. A. (2007). Experimental Anti-Tumor Therapy in
3-D: Spheroids - Old Hat or New Challenge?. Int. J. Radiat. Biol..

[36] Rousselle P., Beck K. (2013). Laminin 332 Processing Impacts Cellular
Behavior. Cell Adh Migr.

[37] Chang, H. Y. ; Chi, J.-T. ; Dudoit, S. ; Bondre, C. ; Van De Rijn, M. ; Botstein, D. ; Brown, P. O. Diversity, Topographic Differentiation, and Positional Memory in Human Fibroblasts. www.ncbi.nlm.nih.gov. 10.1073/pnas.162488599 PMC130553 12297622

[38] Sivagurunathan S., Vahabikashi A., Yang H., Zhang J., Vazquez K., Rajasundaram D., Politanska Y., Abdala-Valencia H., Notbohm J., Guo M., Adam S. A., Goldman R. D. (2022). Expression
of Vimentin Alters Cell Mechanics, Cell-Cell Adhesion, and Gene Expression
Profiles Suggesting the Induction of a Hybrid EMT in Human Mammary
Epithelial Cells. Front. Cell Dev. Biol..

[39] Krishnamoorthy N., Tseng Y., Gajendrarao P., Sarathchandra P., McCormack A., Carubelli I., Sohier J., Latif N., Chester A. H., Yacoub M. H. (2018). A Strategy
to Enhance Secretion of
Extracellular Matrix Components by Stem Cells: Relevance to Tissue
Engineering. Tissue Eng. Part A.

[40] Zurdo
Schroeder I., Franke P., Schaefer U. F., Lehr C. M. (2007). Development
and Characterization of Film Forming Polymeric Solutions for Skin
Drug Delivery. Eur. J. Pharm. Biopharm..

[41] Zhang H., Li L. L., Dai F. Y., Zhang H. H., Ni B., Zhou W., Yang X., Wu Y. Z. (2012). Preparation and
Characterization of Silk Fibroin as a Biomaterial with Potential for
Drug Delivery. J. Transl. Med..

[42] Hu X., Kaplan D., Cebe P. (2006). Determining Beta-Sheet Crystallinity
in Fibrous Proteins by Thermal Analysis and Infrared Spectroscopy. Macromolecules.

[43] Maurizii G., Moroni S., Jimènez
Núnez J. V., Curzi G., Tiboni M., Aluigi A., Casettari L. (2024). Non-Invasive
Peptides Delivery Using Chitosan Nanoparticles Assembled via Scalable
Microfluidic Technology. Carbohydrate Polymer
Technologies and Applications.

[44] Riaz T., Zeeshan R., Zarif F., Ilyas K., Muhammad N., Safi S. Z., Rahim A., Rizvi S. A. A., Rehman I. U. (2018). FTIR Analysis
of Natural and Synthetic Collagen. Appl. Spectrosc
Rev..

[45] Çalamak S., Erdoǧdu C., Özalp M., Ulubayram K. (2014). Silk Fibroin
Based Antibacterial Bionanotextiles as Wound Dressing Materials. Materials Science and Engineering C.

[46] Thu H. E., Zulfakar M. H., Ng S. F. (2012). Alginate
Based Bilayer Hydrocolloid
Films as Potential Slow-Release Modern Wound Dressing. Int. J. Pharm..

[47] Maiolo J. R., Ferrer M., Ottinger E. A. (2005). Effects
of Cargo Molecules on the
Cellular Uptake of Arginine-Rich Cell-Penetrating Peptides. Biochimica et Biophysica Acta (BBA) - Biomembranes.

[48] Melikov K., Hara A., Yamoah K., Zaitseva E., Zaitsev E., Chernomordik L. V. (2015). Efficient Entry of Cell-Penetrating Peptide Nona-Arginine
into Adherent Cells Involves a Transient Increase in Intracellular
Calcium. Biochem. J..

[49] Zhang R., Qin X., Kong F., Chen P., Pan G. (2019). Improving Cellular
Uptake of Therapeutic Entities through Interaction with Components
of Cell Membrane. Drug Deliv.

[50] Rousselle C., Clair P., Temsamani J., Scherrmann J.-M. (2002). Improved
Brain Delivery of Benzylpenicillin with a Peptide-Vector-Mediated
Strategy. J. Drug Target.

[51] Sano T., Kume T., Fujimura T., Kawada H., Moriwaki S., Takema Y. (2005). The Formation of Wrinkles
Caused by Transition of Keratin
Intermediate Filaments after Repetitive UVB Exposure. Arch Dermatol Res..

[52] Lee H., Hong Y., Kim M. (2021). Structural
and Functional Changes
and Possible Molecular Mechanisms in Aged Skin. Int. J. Mol. Sci..

[53] Fraser J. R., Laurent T. C., Laurent U. B. (1997). Hyaluronan: Its Nature, Distribution,
Functions and Turnover. J. Intern. Med..

[54] Williams A. C., Barry B. W. (2004). Penetration Enhancers. Adv. Drug
Deliv Rev..

[55] Choe C., Schleusener J., Lademann J., Darvin M. E. (2017). Keratin-Water-NMF
Interaction as a Three Layer Model in the Human Stratum Corneum Using
in Vivo Confocal Raman Microscopy. Sci. Rep..

[56] El
Omri A., Han J., Yamada P., Kawada K., Abdrabbah M. B., Isoda H. (2010). Rosmarinus Officinalis Polyphenols Activate Cholinergic Activities
in PC12 Cells through Phosphorylation of ERK1/2. J. Ethnopharmacol..

[57] Teleanu R. I., Niculescu A.-G., Roza E., Vladâcenco O., Grumezescu A. M., Teleanu D. M. (2022). Neurotransmitters—Key Factors
in Neurological and Neurodegenerative Disorders of the Central Nervous
System. Int. J. Mol. Sci..

[58] Dobransky T., Rylett R. J. (2005). A Model for Dynamic
Regulation of Choline Acetyltransferase
by Phosphorylation. J. Neurochem..

[59] Blanes-Mira C., Clemente J., Jodas G., Gil A., Fernández-Ballester G., Ponsati B., Gutierrez L., Pérez-Payá E., Ferrer-Montiel A. (2002). A Synthetic
Hexapeptide (Argireline) with Antiwrinkle
Activity. Int. J. Cosmet Sci..

[60] Treiber N., Maity P., Singh K., Ferchiu F., Wlaschek M., Scharffetter-Kochanek K. (2012). The Role of
Manganese Superoxide
Dismutase in Skin Aging. Dermatoendocrinol.

[61] Wang Z., Yan Y., Zhang Z., Li C., Mei L., Hou R., Liu X., Jiang H. (2024). Effect of
Chitosan and Its Water-Soluble Derivatives
on Antioxidant Activity. Polymers (Basel).

[62] Cao C., Xiao Z., Tong H., Liu Y., Wu Y., Ge C. (2022). Oral Intake of Chicken Bone Collagen
Peptides Anti-Skin Aging in
Mice by Regulating Collagen Degradation and Synthesis, Inhibiting
Inflammation and Activating Lysosomes. Nutrients.

[63] Kalyanaraman B., Darley-Usmar V., Davies K. J. A., Dennery P. A., Forman H. J., Grisham M. B., Mann G. E., Moore K., Roberts L. J., Ischiropoulos H. (2012). Measuring
Reactive Oxygen and Nitrogen Species with
Fluorescent Probes: Challenges and Limitations. Free Radic Biol. Med..

[64] Rinnerthaler M., Bischof J., Streubel M., Trost A., Richter K. (2015). Oxidative
Stress in Aging Human Skin. Biomolecules.

[65] Zeichner J., Bussmann T., Weise J. M., Maass E., Krüger A., Schade A.-K., Lain E., Mariwalla K., Kirchner F., Draelos Z. D. (2024). Evaluation of Antioxidants’
Ability to Enhance Hyaluronic-Acid Based Topical Moisturizers. J. Clin. Aesthet. Dermatol..

[66] Lin Q., Song B., Zhong Y., Yin H., Li Z., Wang Z., Cheong K.-L., Huang R., Zhong S. (2023). Effect of
Sodium Hyaluronate on Antioxidant and Anti-Ageing Activities in Caenorhabditis
Elegans. Foods.

[67] Varani J., Warner R. L., Gharaee-Kermani M., Phan S. H., Kang S., Chung J., Wang Z., Datta S. C., Fisher G. J., Voorhees J. J. (2000). Vitamin A Antagonizes
Decreased Cell Growth and Elevated
Collagen-Degrading Matrix Metalloproteinases and Stimulates Collagen
Accumulation in Naturally Aged Human Skin1. Journal of Investigative Dermatology.

[68] Gao J., Guo Z., Zhang Y., Liu Y., Xing F., Wang J., Luo X., Kong Y., Zhang G. (2023). Age-Related Changes in the Ratio
of Type I/III Collagen and Fibril Diameter in Mouse Skin. Regen. Biomater..

[69] Wang Y., Wang M., Xiao S., Pan P., Li P., Huo J. (2013). The Anti-Wrinkle Efficacy of Argireline,
a Synthetic Hexapeptide,
in Chinese Subjects. Am. J. Clin Dermatol.

[70] Sorushanova A., Delgado L. M., Wu Z., Shologu N., Kshirsagar A., Raghunath R., Mullen A. M., Bayon Y., Pandit A., Raghunath M., Zeugolis D. I. (2019). The Collagen Suprafamily: From Biosynthesis
to Advanced Biomaterial Development. Adv. Mater..

