# Supplementary material for: Development of a Sensory Neuron-Integrated Skin Spheroid Model for the Evaluation of Neuropeptide-Based Topical Delivery Systems
Source: ACS Biomater Sci Eng. 2025 May 23;11(6):3503–22. doi: 10.1021/acsbiomaterials.5c00141 (PMC12818723; doi:10.1021/acsbiomaterials.5c00141)
Supplement: Supplementary file 1 [file ab5c00141_si_001.pdf]

Development of a Sensory Neuron-Integrated Skin Spheroid Model for the Evaluation of Neuropeptide-Based Topical Delivery Systems

Bianca Aparecida Martin[1], Juliana Viegas[2], Luciana Facco Dalmolin[1], Emerson de Souza Santos[1], Izabela Pereira Vatanabe[1], Sabrina Francesca Lisboa[1], Renata F. V. Lopez[1] and Bruno Sarmento[2,3,4]*

[1] School of Pharmaceutical Sciences of Ribeirão Preto, University of São Paulo, Avenida do Café, s/nº, 14040-903 Ribeirão Preto, São Paulo, Brazil

[2] i3S – Instituto de Investigação e Inovação em Saúde, Universidade do Porto, Rua Alfredo Allen 208, 4200-135 Porto, Portugal

[3] INEB – Instituto de Engenharia Biomédica, Universidade do Porto, Rua Alfredo Allen 208, 4200-135 Porto, Portugal

[4] IUCS-CESPU – Instituto Universitário de Ciências da Saúde, Rua Central de Gandra 1317, 4585-116 Gandra, Portugal

Supporting information

Table S1. List of antibodies and staining used for immunofluorescence imaging of SS and EDS.

**Specification**	**Target**	**Supplier**	**Reference**	**Host**	**Target species**	**Dilution used**
Primary	β-tubulin III	Promega Corporation	G712A	Mouse	Human	1:2000
Primary	NeuN	Abcam	ab177487	Rabbit	Mouse, Rat, Human, Zebrafish, Common marmoset, Dog, Cat, Goat and Sheep	1:500
Primary	Choline acetyltransferase (ChAT)	Merck	AB144P	Goat	Human, Rat, Mouse, Monkey, Opossum, Avian, Chicken, Guinea Pig and Zebrafish	1:100
Primary	Fibronectin	Sigma-Aldrich	F3648	Rabbit	Human	1:400
Primary	Vimentin	Santa Cruz Biotecnology	sc-6260	Mouse	Mouse, Rat and Human	1:200
Primary	Laminin	Sigma-Aldrich	L9393	Rabbit	Human	1:50
Primary	Collagen I	Rockland	600-401-103-0.1	Rabbit	Mouse, Human, Rat, Bovine and Pig	1:100
Secondary	Alexa Fluor™ 647	Invitrogen	A-21235	Goat	Mouse	1:500
Secondary	Alexa Fluor ™ 594	Invitrogen	A-11005	Goat	Rabbit	1:1000
Staining	Phalloidin (Alexa Fluor ™ 546)	ThermoFisher	A22283	-	-	1:2000
Staining	Phalloidin (Alexa Fluor™ 488)	ThermoFisher	A12379	-	-	1:2000 and 1:225
Staining	DAPI	Merck	D9542	-	-	500 ng/mL and 1:1000

Table S2. Scores attributed to the observed effects on the chorioallantoic membrane as a function of time after treatment with the films. Source: Adapted from[13].

Time (T)	Score		
	Hyperemia	Hemorrhage	Coagulation
$T \leq 30$ s	5	7	9
30 s < $T \leq 2$ min	3	5	7
2 min < $T \leq 5$ min	1	3	5

Table S3. Primers used in qRT-PCR amplification.

Gene	Name	Sequence (5'->3')	Tm (°C)	Amplikon length (bp)
SOD2	hSOD2aF	TCCAGGCAGAAGCACAG	61	140
	hSOD2aR	TTCTCCTCGGTGACGTTC	61	
ACTN1	hACTN1aF	AGAAATCGTGGATGGGAATG	60	190
	hACTN1aR	GCCATCCTTCCAGCTTATG	60	

Table S4. Classification of the effects of films with and without HEX-3 in the HET-CAM assay after 5 minutes of contact, according to[13]. Sodium Lauryl Sulfate 1% (SDS) was the positive control, while Sodium Chloride 0.9% (NaCl) was the negative control. Values expressed as mean ± standard deviation (SD) (n=4).

Treatment	Irritation Score	Classification
SDS 1%	12 ± 0	Severe irritant
NaCl 0.9 %	0 ± 0	Not irritating
Blank film	1.25 ± 2.5	Mild irritant
HEX3-film	1.5 ± 2.4	Mild irritant

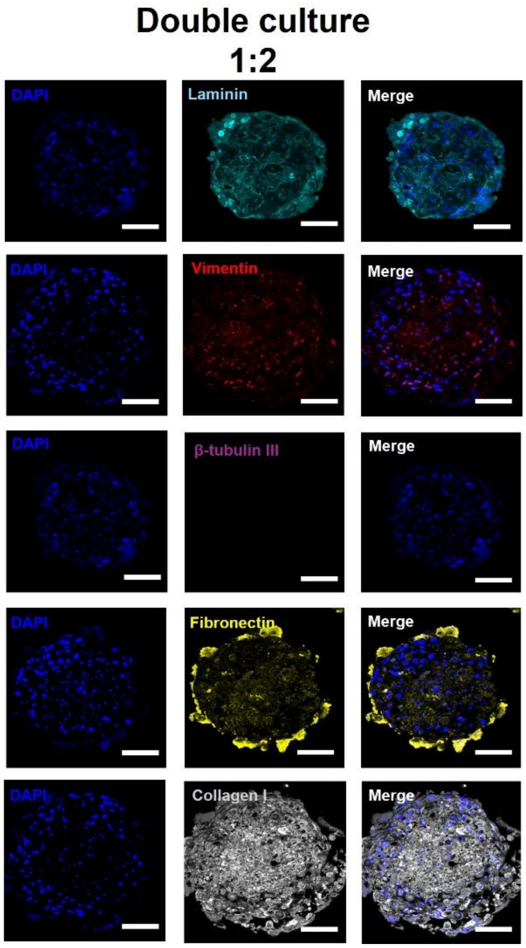

Figure S1. Immunofluorescence micrographs of representative histological sections of SS spheroids in double culture at a ratio of 1:2 (HaCaT:HDF). Representative images of the cellular organization of spheroids: Laminin (cyan); Vimentin (red); β-tubulin III (violet); Fibronectin (yellow) and Type I collagen (gray) contrasted with DAPI (blue). Scale bar: 50 μm.

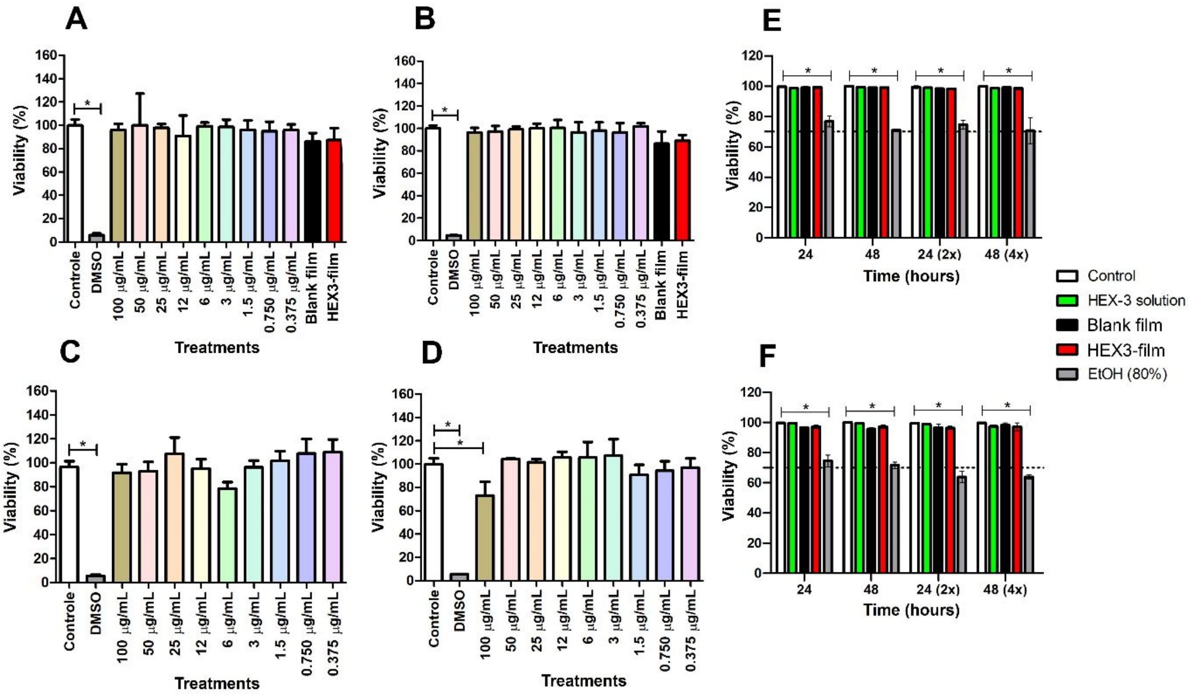

Figure S2. Cell viability of monoculture cells (A) keratinocytes (HaCaT), (B) fibroblasts (HDF), (C) neurons-like (NSC-34) and (D) human mesenchymal stem cells (hMSCs) after treatment with the film containing or not HEX-3 and different concentrations of HEX-3 solution (100 μg/mL - 0.375 μg/mL) (t-test, $p \leq 0.05$ (n=5)). *Significant difference between control and treatment. Cell viability of (E) young and (F) elderly skin treated with the film containing or not HEX-3 and HEX-3 solution (600 ng). EtOH 80% was used negative control (t-test, $p \leq 0.05$, n=3). *Significant

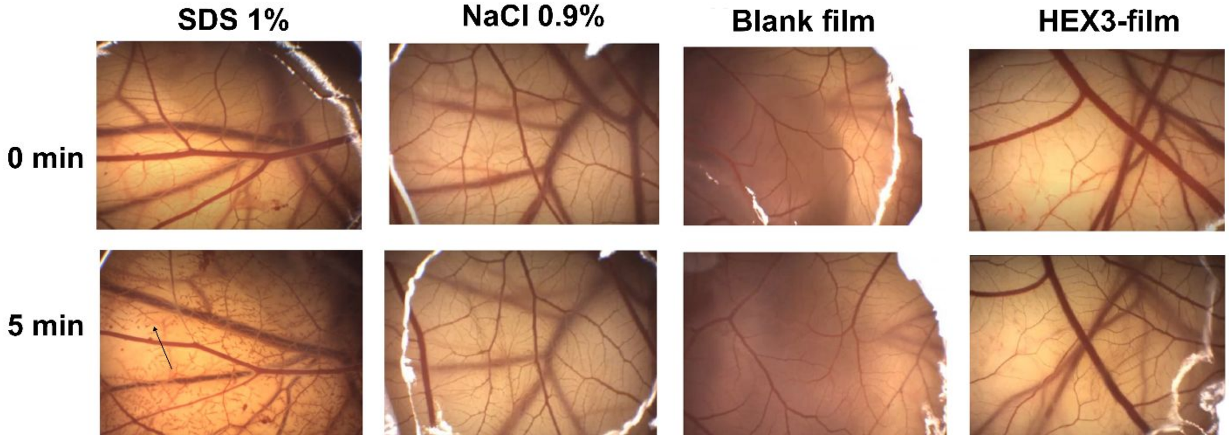

Figure S3. Vascular effects on the chorioallantoic membrane in the HET-CAM assay monitored for 5 minutes after treatment with the Blank and HEX3-films. 1% SDS: Positive control and 0.9% NaCl: Negative control. Black arrow: hemorrhage.

Reference

(13) Luepke, N. P.; Kemper, F. H. *THE HET-CAM TEST: AN ALTERNATIVE TO THE DRAIZE EYE TEST*; 1986; Vol. 24.
